# Cytochrome P450 metabolism of the post-lanosterol intermediates explains enigmas of cholesterol synthesis

**DOI:** 10.1038/srep28462

**Published:** 2016-06-23

**Authors:** Jure Ačimovič, Sandeep Goyal, Rok Košir, Marko Goličnik, Martina Perše, Ales Belič, Žiga Urlep, F. Peter Guengerich, Damjana Rozman

**Affiliations:** 1 Center for Functional Genomics and Bio-Chips, Faculty of Medicine, University of Ljubljana, Zaloška 4, SI-1000 Ljubljana, Slovenia; 2 Institute of Biochemistry, Faculty of Medicine, University of Ljubljana, Vrazov trg 2, SI-1000 Ljubljana, Slovenia; 3 Department of Biochemistry, Vanderbilt University School of Medicine, Nashville, TN 37232-0146, United States; 4 Medical Experimental Centre, Institute of Pathology, Faculty of Medicine, University of Ljubljana, Zaloška 4, SI-1000 Ljubljana, Slovenia; 5 Faculty of Electrical Engineering, University of Ljubljana, Slovenia

## Abstract

Cholesterol synthesis is among the oldest metabolic pathways, consisting of the Bloch and Kandutch-Russell branches. Following lanosterol, sterols of both branches are proposed to be dedicated to cholesterol. We challenge this dogma by mathematical modeling and with experimental evidence. It was not possible to explain the sterol profile of testis in cAMP responsive element modulator tau (*Crem* *τ*) knockout mice with mathematical models based on textbook pathways of cholesterol synthesis. Our model differs in the inclusion of virtual sterol metabolizing enzymes branching from the pathway. We tested the hypothesis that enzymes from the cytochrome P450 (CYP) superfamily can participate in the catalysis of non-classical reactions. We show that CYP enzymes can metabolize multiple sterols *in vitro*, establishing novel branching points of cholesterol synthesis. In conclusion, sterols of cholesterol synthesis can be oxidized further to metabolites not dedicated to production of cholesterol. Additionally, CYP7A1, CYP11A1, CYP27A1, and CYP46A1 are parts of a broader cholesterol synthesis network.

Cholesterol synthesis is a basic pathway believed to take place in virtually all mammalian cells. In this anabolic pathway the cholesterol molecule is built from acetate in over 30 reactions and enzymes from different protein families. The pathway is composed of the isoprenoid synthetic reactions forming squalene (the pre-squalene or pre-lanosterol part), which is well characterized^1^, and the post-squalene phase in which demethylations and reductions of sterol intermediates convert lanosterol to cholesterol. A detailed metabolic reaction network, with information available from the literature and the pathway databases (Kyoto Encyclopedia of Genes and Genomes (KEGG) (http://www.genome.jp/kegg/), BioCyc database collection (http://biocyc.org/), LIPID Metabolites And Pathways Strategy (LIPID MAPS) (http://www.lipidmaps.org/)), is shown in Supplementary Fig. 1^2^,^3^,^4^,^5^,^6^,^7^. Initially the post-squalene pathway was divided into the Bloch and Kandutsch-Russell (K-R) branches^8^,^9^. In the Bloch branch, the final reaction to form cholesterol involves the conversion of desmosterol to cholesterol by sterol-Δ^24^-reductase (DHCR24); thus, all intermediates from lanosterol to desmosterol contain an unsaturated Δ^24^ bond. In contrast, in the K-R branch, DHCR24 acts earlier, on lanosterol; thus all intermediates from 24,25-dihydrolanosterol (DHL) to 7-dehydrocholesterol (7-DHC) contain a saturated side chain. Since DHCR24 can, in principle, metabolize any cholesterol synthesis intermediate from lanosterol on, the two branches are usually combined to produce a textbook cholesterol synthesis pathway^4^. A recent flux analysis of cholesterol synthesis surprisingly revealed that the K–R pathway is not used in tissues and cells *in vivo*^10^. Instead, a hybrid pathway exists where the transition from the Bloch to the K–R pathway depends on the tissue or cell type. Testes apply mainly the Bloch pathway, and the flux of intermediates drops from testis meiosis-activating sterol (T-MAS) to zymosterol. These findings suggested that a large fraction of sterols is diverted from the cholesterol pathway in the testis to produce other, yet unidentified sterols^10^,^11^. Metabolism of lanosterol differs between liver and the testes, as shown in earlier work^12^,^13^,^14^,^15^ and confirmed by the recent flux analysis^10^.

We show here that intermediates of the Bloch and K-R branches leak from the pathway and can be metabolized by enzymes of the cytochrome P450 (CYP) superfamily in the testes of mice with defective post-lanosterol cholesterol synthesis. The leakage was proposed by a mathematical model in which the textbook cholesterol synthesis pathways failed to describe the experimental gene expression and sterol data. In the second iterative cycle, *in vitro* enzyme activity studies identified novel enzymes that catalyse sterol modifications outside the Bloch and K-R pathways, thus confirming the proposed *in silico* branches. The applied mouse model involves a knockout for the transcription factor *Crem,* where males are infertile due to an arrest in spermatogenesis^16^. Among other defects, the mice lack the germ cell-specific lanosterol-14α-demethylase (*Cyp51*) mRNA^15^ producing the germline form of *Cyp51* from the post-lanosterol cholesterol synthesis. T-MAS accumulates in the testes of normal mice due to cAMP responsive element modulator (CREM)-dependent transcriptional activation of *Cyp51*^10^,^17^ and can be formed even in mature sperm^18^,^19^. In this work we provide evidence of novel branching points in the post-lanosterol cholesterol synthesis and reveal new roles of CYP enzymes in cholesterol homeostasis.

## Results

### Gene expression in testis of *Crem* ^−/−^ mice

**Figure 1:**
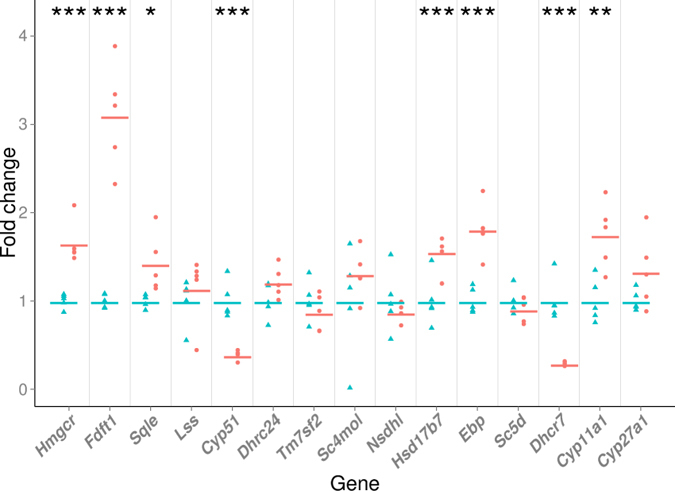
Expression levels of cholesterogenic genes. Expression is represented as fold change of each individual mouse compared to the wild-type (WT) average for each gene separately. Triangles (blue) and circles (red) present WT and *Crem*^−/−^ animals, respectively. Lines represent average expression for each gene according to the genotype. Asterisks present p-values: *** for p < 0.001, ** for p < 0.01, * for p < 0.05. Gene abbreviations are according to Unigene (see Abbreviations in Supplementary information).

The relative expression levels of 15 cholesterogenic genes, *Crem*^−/−^ compared to wild-types (WT), are shown in Fig. 1, with 13 of them being involved in the cholesterol synthesis pathway and two in post-cholesterol synthesis transformations of cholesterol (cytochrome P450-family-11-subfamily-A-polypeptide-1 (*Cyp11a1*) gene and cytochrome P450, family 27, subfamily A, polypeptide 1 (*Cyp27a1*) gene). Triangles (WT) and circles (*Crem*^−/−^) represent fold change of each individual mouse relative expression compared to average WT expression for each cholesterogenic gene separately. Five genes of the cholesterol synthesis pathway showed statistically significant higher expressions in *Crem*^−/−^ compared to WT animals, *Hmgcr* (1.65-fold), *Fdft1* (3.09-fold), *Sqle* (1.42-fold), *Hsd17b7* (1.55-fold) and *Ebp* (1.81-fold), with p-values lower than 0.001, 0.001, 0.05, 0.001, and 0.001, respectively. Only two showed statistically significant lower expression levels, *Cyp51* (0.39-fold) as the proposed direct target of CREM regulation and *Dhcr7* (0.29-fold), with p-values < 0.001. Expression of the six remaining cholesterol synthesis genes (*Lss*, *Dhcr24*, *Tm7sf2*, *Sc4mol*, *Nsdhl,* and *Sc5d*) was unchanged. Expression of two genes involved in the cholesterol metabolism was also evaluated in *Crem*^−/−^ compared to WTs. *Cyp11a1* expression was increased (1.74-fold) while *Cyp27a1* remained unchanged, with p-values of p < 0.01 and p = 0.11, respectively.

### Cholesterol synthesis intermediates in testes of *Crem* ^−/−^ mice

**Figure 2:**
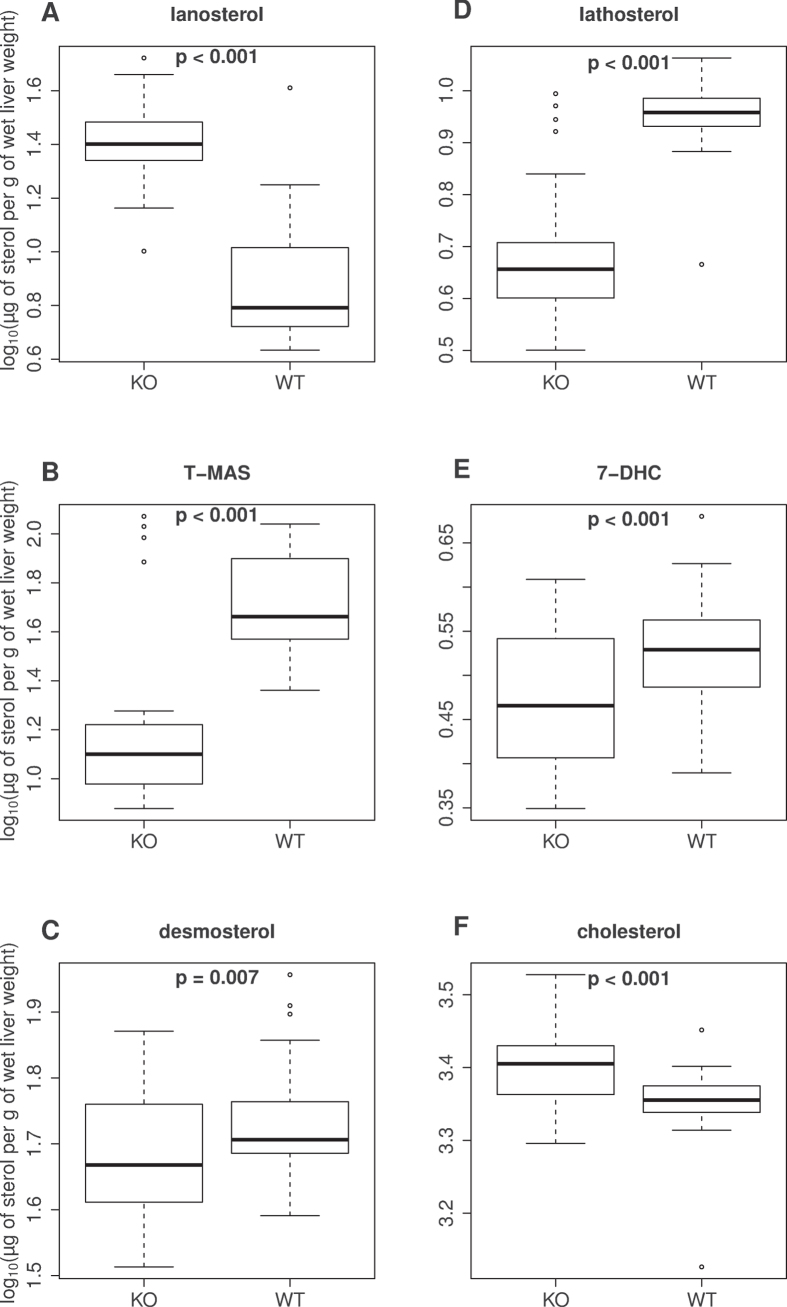
Cholesterol synthesis sterol concentrations. Boxplots represent log_10_(*μ*g/g testes wet weight) measurements of the sterols lanosterol, T-MAS (testis meiosis-activating sterol), lathosterol, 7-DHC (7-dehydrocholesterol), desmosterol, and cholesterol for each genotype (WT, wild-type; KO, *Crem*^−/−^) with associated p-values.

Log_10_(μg/g testes wet weight) values for sterol intermediate concentrations are presented in Fig. 2 as boxplots, showing that lanosterol was significantly higher (p < 0.001, as expected due to the lower expression of *Cyp51* gene, T-MAS (p < 0.001), lathosterol (p < 0.001), 7-DHC (p < 0.001) and desmosterol (p = 0.007) were significantly lower and the final product of the synthesis pathway, cholesterol, was significantly higher (p < 0.001) in mouse testes of 33 *Crem*^−/−^ compared to 42 WT animals, adjusted on the bases of animal age and experiment date. Interestingly, lanosterol was highly accumulated (3.28-fold increase) while T-MAS (0.26-fold) and lathosterol (0.53-fold) show the largest drops in the *Crem*^−/−^ testis. Desmosterol and 7-DHC, the immediate precursors of cholesterol were slightly (0.88-fold and 0.86-fold) diminished in the *Crem*^−/−^ while cholesterol was slightly elevated (1.15-fold).

### Simulation of the cholesterol synthesis network model proposed virtual enzymes that metabolize post-lanosterol sterols

**Figure 3:**
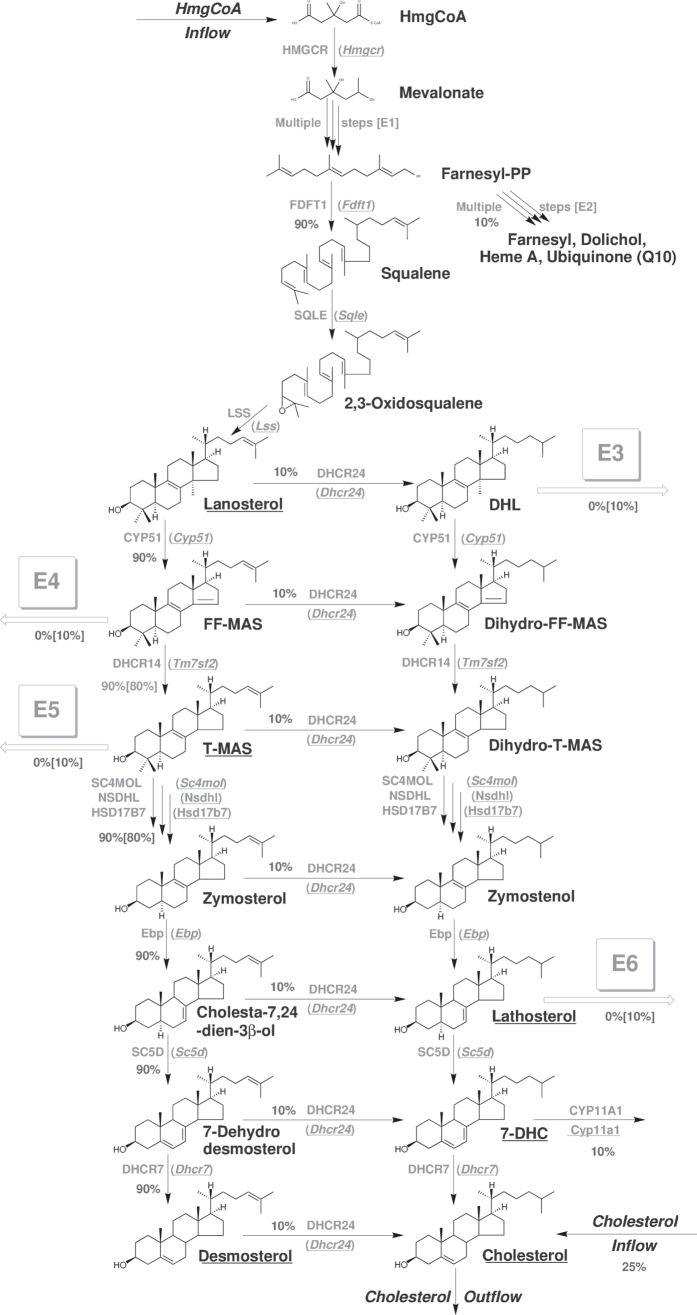
The cholesterol synthesis pathway, combined with a mathematical model. The underlined genes (gray) and sterol intermediates (black) were measured in the present study. Enzyme (gene) abbreviations are according to Unigene (see Abbreviations in Supplementary information). E1 and E2 present multiple enzymes combined, while E3, E4, E5 and E6 present minimal requirements for mathematical model illustration of the sterol intermediate levels measured. Numbers (%) show initial division of metabolic flux at branching points, while numbers in squared brackets show final model division values. DHL, 24,25-dihydrolanosterol; FF-MAS, follicular-fluid meiosis-activating sterol; T-MAS, testis meiosis-activating sterol; 7-DHC, 7-dehydrocholesterol.

The simulation of the initial (textbook) model (Fig. S1) showed significant differences between the simulated and actual metabolite levels. Stepwise modifications of the model were performed in order to correlate the experimental and simulated data (see Supporting Information). **Initial model 0.** The initial (textbook) model was used without modifications and relative gene expressions were directly translated to the relative enzyme activities. The relative metabolite concentrations are presented in Fig. S2. The model did not describe the observed situation well because levels of several metabolites were much higher in the model. In the first model correction (**model 1**), the enzyme level changes were assumed to correlate perfectly with the corresponding mRNA levels for the late part of the cholesterol synthesis; however, levels of 3-hydroxy-3-methylglutaryl-CoA reductase (HMGCR) were optimized, as described in the Supplementary Information. These modifications resulted in an optimal fit of simulated and measured data for lanosterol, but the fit for other metabolites was as poor as in the initial model settings (Fig. S3). The resulting gene expressions and enzyme activities are presented in Fig. S4. We can observe only smaller HMGCR activity in comparison with its expression. In the second model correction (**model 2**), activities of all cholesterogenic enzymes were optimized in a similar way as for HMGCR. First the optimal solution with no limitation to enzyme activity was found and in the next step the optimal solution was found where the maximal relative gene activity was limited with the relative gene expression of the corresponding genes which is physiologically more acceptable. The results are shown in Figs S5–S8. The results show that it is possible to fit the relative metabolite levels when no limits are set on the enzyme activities; however, substantial improvement of the criterion function with respect to the model where no enzyme activities were adapted was still achieved even with the limited adaptation where the relative enzyme activities were limited below the relative gene expression. The solution is not acceptable in terms of measurement error of the metabolites. For the third modification (**model 3**), we again assumed total correlation of the enzyme level with changes of the corresponding mRNA levels changes for the late part of cholesterol synthesis, while the flux distributions (Fig. 3) were optimized. The relative metabolite levels are shown in Fig. S9, initial and optimal flux distributions are shown in Fig. S10. The optimized initial flux distribution of WT has substantially improved the model fit; however, the new flux distribution is not physiologically sound since after T-MAS the majority of the flux switches from Bloch branch to K-R branch of the network, which is not consistent with the literature data. As aforementioned, both experiments introduced insignificant improvements to the fit. This lead to the hypothesis that intermediates of cholesterol synthesis are likely eliminated from the pathway by branching enzymes. In the fourth model correction (**model 4**), we introduced a branch pathway that eliminated 10% of the steady-state flux of DHL (Fig. 3, enzyme E3) for WT while HMGCR and the levels of enzymes of the late part of the pathway, including E3 for DHL elimination, were optimized and the initial distribution of metabolic fluxes was used.

Adaptation schemes with limited values of enzyme activities were applied since with the unlimited optimization even the simpler model 2 can fit the metabolite profile. The results are presented in Figs S11 and S12. The model shows no improvement with respect to model 2 in terms of metabolite profile fit; however, relative gene expression of *Cyp51* is closer to its activity. The simulated metabolite profiles are still not within the measurement errors for metabolites. The fifth modification (**model 5**) was the addition of the lathosterol elimination to the fourth model modification (Fig. 3, enzyme E6), conveying 10% of the steady-state flux of lathosterol for WT and optimized with the rest of the enzyme levels, as in the fourth experiment. Adaptation schemes with limited values of enzyme activities were applied. Results are presented in Figs S13 and S14. The model shows some minor improvement with respect to the model 4, however, the simulated metabolite profiles were still not within the measurement errors for metabolites. In the sixth correction (**model 6**), three elimination enzymes (E3 for DHL, E4 for follicular-fluid meiosis-activating sterol (FF-MAS), and E6 for lathosterol) were added to the textbook model and all enzyme activities were adapted to find best fit of the metabolite profile. Adaptation schemes with limited values of enzyme activities were applied. These results are presented in Figs S15 and S16. The model showed some minor improvement with respect to model 5: however, the metabolite profiles are still not within the measurement errors for metabolites and activity of enzyme E3 is over 8-fold higher for *Crem*^−/−^ than WT, which is rather unlikely in a real system.

**Figure 4:**
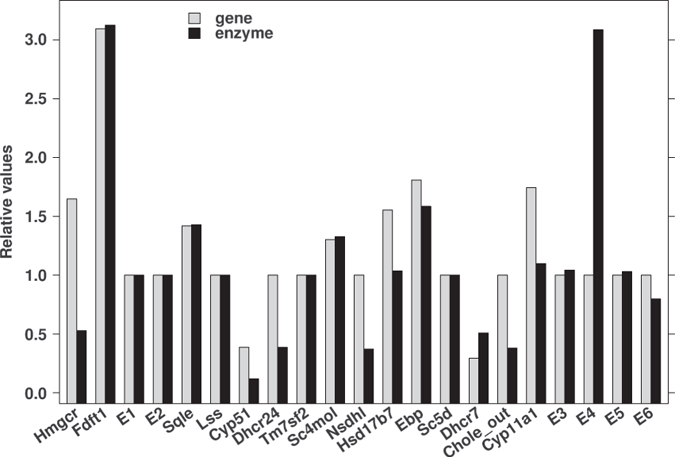
Optimization of enzyme levels (Model: Experiment seven). Optimization results for enzyme levels (black) compared to experimental mRNA levels (gray) for experiment seven of the simulation. E3–E6 represent the virtual enzymes. mRNA levels are set to value 1.00.

Therefore, the **seventh and final model modification** (model 7 – final model) was introduced to correct for this anomaly, adding the fourth virtual enzyme E5 to eliminate T-MAS form the synthetic pathway on top of previously described novel elimination paths (E3, E4 and E6). Adaptation schemes with limited values of enzyme activities were applied. These results are presented in Figs S17 and S18. The latter model (**model seven**) showed an ideal fit of the measured and simulated levels of metabolites (Table 1), and enzyme optimization results are shown in Fig. 4.

The following simulation experiments were designed to: (a) determine whether all elimination enzymes (E3–E6) are necessary for adequacy of the model that gave us the best fit, and b) validate the possibility that a single enzyme (E3–E6 combined into one) may be involved in the process of elimination; however, we were not able to fit the metabolite levels with the model. Finally, we performed sensitivity analysis on **model seven**, where the optimal metabolite levels were evaluated with respect to the enzyme levels. The levels of enzymes that were subject to optimization were simultaneously varied within 5% of the optimal values. A random population of 10,000 enzyme levels, normally distributed around their optimal values, was generated and simulated. The resulting population of 10,000 corresponding metabolite levels also showed normal distributions, with standard deviations between 4 and 7% of the optimal levels. The distribution of the criterion value J for the experiment was skewed with a median value of 0.13, steeply descending towards 0, suggesting a relatively narrow minimum of the criterion function and, consecutively, a narrow range of optimal enzyme levels indicative of a well-defined optimization problem.

Only the model with elimination paths of DHL (E3), lathosterol (E6), FF-MAS (E4), and T-MAS (E5) can describe the measured metabolite ratios between *Crem*^−/−^ and WT with sufficient precision and with physiological acceptable differences between simulated enzyme activities and measured gene expressions.

### Sterols from cholesterol synthesis are metabolized by a variety of CYP enzymes

Post-lanosterol intermediates from the Bloch and the K-R pathways were tested as substrates with four human CYP enzymes that are known to oxidize cholesterol. A summary of all products identified by comparison with authentic standards, by gas chromatography-mass spectrometry (GC-MS) fragmentation or by NMR, is given in Table 2. Of eleven sterols used as substrates, we were able to definitively identify products from five (7-DHC, zymostenol, lathosterol, desmosterol, and cholesterol). In the remaining cases, the levels of products were either too low to be practical for characterization or identification was not successful, so we provide only the percentages of substrate conversion (Table 3).

Surprisingly, most post-lanosterol intermediates are substrates for cytochrome P450, family 7, subfamily A, polypeptide 1 (CYP7A1), CYP27A1, and cytochrome P450, family 46, subfamily A, polypeptide 1 (CYP46A1) *in vitro.* The side chain cleavage enzyme CYP11A1 is more specific: only 7-dehydrocholesterol and desmosterol, the immediate precursors of cholesterol, were substrates for this enzyme (Table 3).

CYP7A1, CYP11A1, CYP27A1, and CYP46A1 converted desmosterol to 7*α*-hydroxydesmosterol, pregnenolone, 27-hydroxydesmosterol, and 24*S*,25-epoxycholesterol and 27-hydroxydesmosterol, respectively.

**Figure 6:**
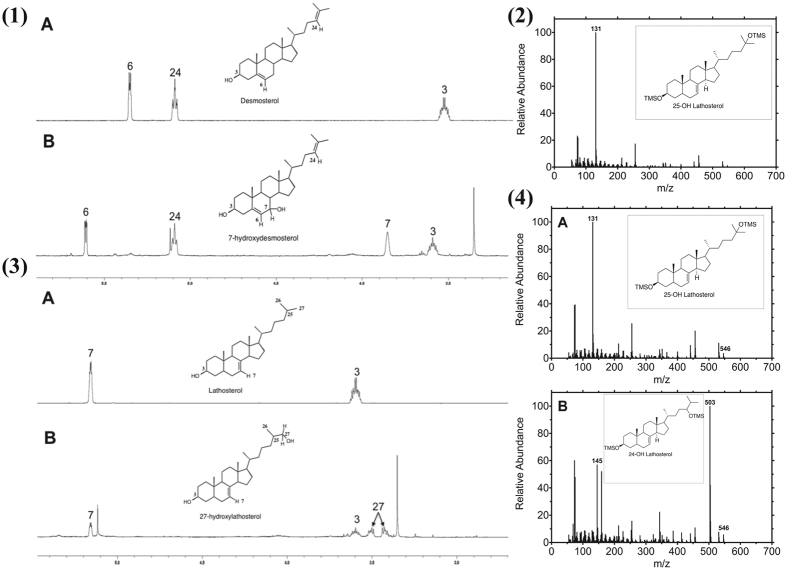
Identification of sterol products. (1) ^1^H NMR spectra of (**A**) desmosterol and (**B**) purified product obtained from reaction of CYP7A1 and desmosterol. (2) GC-MS fragmentation of the product (TMS ether derivative) obtained from reaction of lathosterol with CYP27A1. (3) ^1^H NMR spectra of (**A**) lathosterol and (**B**) purified product obtained from reaction of CYP27A1 with lathosterol. (4). GC-MS fragmentation of the product (TMS ether derivative) obtained from reaction of lathosterol with CYP46A1. The fragment *m*/*z* 131 is indicative of loss of the elements of C(CH_3_)_2_OSi(CH_3_)_3_, consistent with hydroxylation at C25 (parts 2,4A). The fragment *m*/*z* 145 (addition of one methylene) is indicative of loss of the elements of (CH_3_)_2_CH_2_COSi(CH_3_)_3_, consistent with hydroxylation at C24 (part 4B). These fragmentation patterns (base peaks) were matched with standard 24- and 25-hydroxycholesterol (data not shown) with the assumption that a change in the position of double bond in “B” ring of sterols would not have any effect on the *α*-cleavage of an TMS isopropyl ether of the side chain, which is far from the “B” ring of the sterol.

Incubation of CYP7A1 with desmosterol yielded one product in the liquid chromatography-mass spectrometry (LC-MS) profile (Fig. 5.1). In the ^1^H NMR spectrum one new peak was present (compared to the substrate desmosterol) at *δ* 3.86 ppm with integration to one proton, indicative of a –CH group (Fig. 6.1), confirmed by heteronuclear single-quantum correlation (NMR) spectroscopy (HSQC) (Fig. S19). Correlation (NMR) spectroscopy (COSY) showed that the protons at *δ* 3.86 and 5.6 ppm are coupled to each other, indicating that these protons are on adjacent carbons (Fig. 6, Figs S19 and S20). The product was identified from ^1^H NMR (Fig. 6, Figs S19 and S20) and ^13^C NMR (not shown) as 7*α*-hydroxydesmosterol, based on chemical shifts of the H7 and H6 protons.

Incubation of CYP11A1 with desmosterol or 7-dehydrocholesterol yielded one product in the LC-MS profile in each case. CYP11A1 cleaves the side chain of cholesterol in a three-step reaction, resulting in pregnenolone. The same product is expected starting from desmosterol. The product (*t*_R_ 1.43 min, Fig. 5.2) was identified as pregnenolone by comparison with an authentic standard.

Incubation of CYP27A1 with lathosterol, 7-DHC, or zymostenol yielded two products in the LC-MS profile in each case (results not shown), while desmosterol yielded only a single product (Fig. 5.3). The minor product from lathosterol was identified as 25-hydroxylathosterol using GC-MS (Fig. 6.2). GC-MS fragmentation of a TMS ether showed a major peak at *m*/*z* 131, characteristic of a 25-hydroxy product (*vide infra*). Similarly, fragmentation analysis of the products formed from 7-dehydrocholesterol and zymostenol yielded 25-hydroxy-7-dehydrocholesterol and 25-hydroxyzymostenol, respectively. The major product formed from lathosterol was identified as 27-hydroxylathosterol by NMR (Fig. 6.3). The two protons attached at C27 (H27) appear to be split, although they are attached to the same carbon atom (C27), because of their diastereotopic nature. In the ^1^H NMR spectrum (compared with the lathosterol substrate) there were two new peaks at *δ* 3.42 and 3.50 ppm, with integration to one proton each (Fig. 6.3), indicative of either two –CH or one –CH_2_ group(s). HSQC NMR confirmed that these protons are attached to a methylene group (CH_2_) and were also split in that spectrum (Fig. S21). The observed splitting patterns in both ^1^H and HSQC NMR matched with the spectra of standard 27-hydroxycholesterol, confirming the product as 27-hydroxylathosterol. Similarly, the NMR spectra of the products derived from 7-dehydrocholesterol and zymostenol confirmed the structures of these products as 27-hydroxy-7-dehydrocholesterol and 27-hydroxyzymostenol, respectively. The product formed from desmosterol (*t*_R_ = 3.97 min, Fig. 5.3) was identified as 27-hydroxydesmosterol by comparison of the LC-MS profile with that obtained from oxidation of desmosterol by CYP46A1 (*t*_R_ = 3.94 min, Fig. 5.4), which was identified as 27-hydroxydesmosterol in our earlier work^20^.

Incubation of CYP46A1 with lathosterol, 7-dehydrocholesterol, zymostenol (data not shown), or desmosterol (Fig. 5.4) yielded two products in the LC-MS profile. The two products from lathosterol were identified as 24-hydroxy- and 25-hydroxylathosterol using GC-MS (Fig. 6). GC-MS fragmentation showed major peaks at *m*/*z* 145 and 503 for the 24-hydroxy product and at *m*/*z* 131 for the 25-hydroxy product (Fig. 6.4), characteristic of 24-hydroxy and 25-hydroxy products. These fragmentation patterns matched those of standard 24- and 25-hydroxycholesterol (data not shown). Similarly, fragmentation of the TMS derivatives of products led to the characterization of 24- and 25-hydroxy-7-dehydrocholesterol formed from 7-dehydrocholesterol and 24- and 25-hydroxyzymostenol as products of zymostenol. The two products from desmosterol were identified as 27-hydroxydesmosterol and 24*S*,25-epoxycholesterol in our earlier work^20^.

## Discussion

Cholesterol is an important constituent of mammalian cell membranes. It is also the precursor of various steroid hormones, e.g. cortisol, aldosterone, progesterone, androgens, and estrogens. About one-fourth of cholesterol arises from dietary intake and about three-fourths from endogenous synthesis^21^. Therefore, the cholesterol synthesis pathway is of considerable biological importance in reproductive organs and is orchestrated by the cAMP-dependent signaling pathway in mouse testes^22^,^23^.

We previously observed that the negative cholesterol feedback regulation mediated by transcription factors of the sterol regulatory element binding transcription factor (SREBF) family is not sufficient to explain the behaviour of the cholesterol synthesis network under various physiological conditions. We proposed that the modulation of post-lanosterol cholesterol synthesis requires interactions between cAMP signaling and cholesterol feedback regulation in both testes^15^ and in somatic cells^24^. In the present study, we propose for the first time that the role of cAMP signaling might be to activate the ‘branching’ metabolism of cholesterol synthesis intermediates when the synthesis pathway is not coupled. This assumption was deduced from mRNA and sterol metabolite measurements in *Crem*^−/−^ mice compared to wild-type, coupled with modeling of the post-squalene cholesterol synthesis pathway^25^, which is distinct from modeling cholesterol synthesis focused on HMGCR as the major regulatory point^26^. Our model proposed branches from the main cholesterol synthesis pathway, described by virtual enzymes E3–E6. The inclusion of CYP11A1 and the virtual enzymes (E3–E6) to the model shows an ideal fit with the measured metabolites (Table 1), where optimization of enzyme levels was based on measured mRNA levels of cholesterogenic genes. Model optimization of HMGCR showed a decrease to 0.5-fold in enzyme expression in *Crem*^−/−^ compared to WT animals, while the experimental mRNA level was increased 1.65-fold. This result can be explained on the basis of previous *in vitro* and *in vivo* studies showing that lanosterol, the first sterol intermediate in cholesterol synthesis, potently stimulates ubiquitination and consequent degradation of HMGCR, whereas cholesterol has no effect^27^. Due to the diminished activity of the lanosterol 14*α*-demethylase in *Crem*^−/−^ mice^15^, lanosterol accumulates in the testes and can lead to an increase in HMGCR degradation. Furthermore, the lanosterol product 27-hydroxylanosterol is even more potent in accelerating HMGCR degradation^27^.

We hypothesize that enzymes E3–E6 are CYP enzymes that metabolize cholesterol and some other sterols, representing new biologically relevant branches from the late portion of cholesterol synthesis. An established branch point is the side chain cleavage enzyme CYP11A1, best known for its initial step in catalysis of steroidogenesis in adrenals and gonads through cAMP-mediated signals^28^,^29^,^30^. In addition, CYP11A1 is important also in vitamin D metabolism and can metabolize 7-dehydrocholesterol to 7-dehydropregnenolone^3^. Our measurements showed significantly higher expression of *Cyp11a1* in testes of *Crem*^−/−^ mice compared to controls, and the branching reaction of CYP11A1 from 7-DHC was accordingly added to the model. The enzyme activity assays show that desmosterol can also be converted to pregnenolone by CYP11A1 as another branch from the cholesterol pathway towards steroid hormones. Even though this reaction has been proposed previously^31^, evidence that this exists has been presented only during this past year by us (in this paper) and others^32^. Based on our data, CYP11A1 removes the sterol side chain only from 7-dehydrocholesterol, desmosterol, and the typical substrate cholesterol (Tables 2 and 3), in contrast with molecular modeling predictions about a much broader substrate specificity^32^.

The second branching candidate enzyme is CYP46A1, initially thought to be expressed mainly in the brain. Recent entries in the Human Protein Atlas show that it is expressed in 41 of 80 tissue cell types analyzed, including brain and testis (http://www.proteinatlas.org/). Interestingly, in the mouse testis the *Cyp46a1* mRNA level was below the detection level. CYP46A1 has a potentially broad substrate specificity for ring-modified sterols. The reported natural substrates (other than cholesterol) are 7-DHC^20^,^33^ and desmosterol^20^. We show that the majority of sterol intermediates in cholesterol synthesis are substrates for CYP46A1, with the exceptions of lanosterol and DHL (Tables 2 and 3).

CYP27A1 is the next proposed branching enzyme. Initially it was characterized in terms of roles in bile acid synthesis, but later a much broader substrate specificity was found^34^. We showed that all sterols tested are oxidized by CYP27A1 (Table 3), being converted to 25- and 27-hydroxy derivatives, when product identification was possible (Table 2). Thus, CYP27A1 is a “general” sterol metabolizing enzyme, but we have not addressed the question of whether all 27-hydroxylated sterols can be metabolized to bile acids.

The last enzyme tested, CYP7A1, is not relevant for the testis but can represent a new branching enzyme in the liver, where it is expressed rather exclusively. It was shown previously that CYP7A1 is active towards desmosterol^35^, lathosterol, and 7-DHC^36^. Similarly, as shown for CYP46A1, the majority of sterols involved in cholesterol synthesis are substrates also for CYP7A1, with the exception of lanosterol and DHL (Tables 2 and 3).

**Figure 7:**
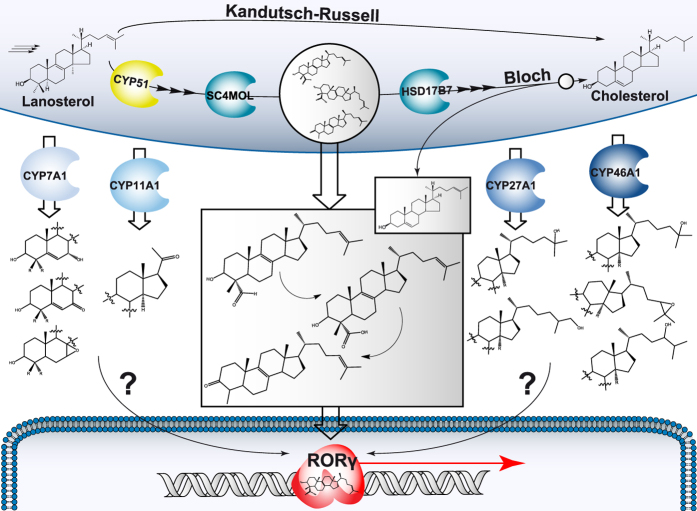
Interplay between cholesterol biosynthesis and RORγ pathway. Cholesterol biosynthesis intermediates downstream of lanosterol have recently been proposed to act as endogenous RORγ ligands. Analysis of the transformation of several cholesterol intermediates with four CYP enzymes revealed potential novel metabolites. These compounds have increased polarity due to the addition of a keto or a hydroxyl group to either the main sterol ring or to the side chain. Although they have yet to be characterized with regards to their RORγ activating potential, the results of Santori *et al*.^37^ indicate that the addition of hydroxyl groups to positions 7, 24, 25, and 27 do not abolish ROR*γ* specific activity.

Due to the recent discoveries, we questioned what the relationship is between sterols downstream of lanosterol, resulting from the herein described CYP metabolism, and the ones identified as potential RAR-related orphan receptor gamma (RORγ) ligands^37^. The general structure of endogenous RORγ ligands^37^,^38^ contains a 3β-hydroxyl or a carbonyl moiety at C3, a possible methyl group at C4 (preferably in β conformation) or a carboxyl group preferred at C4α, and double bonds at either C5 or C8 of the sterol ring and C25 (26) of the side chain (Fig. 7). We have shown that CYP27A1 oxidizes most sterols from the Bloch and the K-R pathways (Table 3) by attaching a hydroxyl group at C25 or C27 of the sterol side-chain. Compared to cholesterol, 25-hydroxycholesterol and 7*α*,27-hydroxycholesterol are potent RORγ activators at low concentrations^37^. Also CYP7A1 metabolites (7*α*-hydroxysterols) and CYP46A1 metabolites (addition of a hydroxyl group to C24 or C25 or an epoxy group) could activate RORγ^37^. While the substrate specificity of CYP enzymes differs for the sterol intermediates (and is generally lower compared to their canonical substrates), the flux through the cholesterol pathway can change dramatically under pathological conditions. For example, in *Cyp51* liver conditional knockout mice with pathology similar to non-alcoholic hepatitis, the CYP51 substrates lanosterol and DHL accumulate while sterols downstream the pathway, such as zymosterol and other potential RORγ natural ligands, are diminished^39^. Increased concentrations of lanosterol and its metabolites might, in part, be responsible for reconstitution of RORγ activity that remained in *Cyp51*^−/−^ fibroblasts despite a complete *Cyp51* ablation^37^,^40^.

## Conclusions

Mathematical modeling of cholesterol synthesis led to the proposal of virtual enzymes branching from the post-lanosterol pathway to explain the experimental data. Activity measurements identified CYP27A1 as the most likely candidate for virtual enzymes E3–E6 in the testis. CYP27A1 metabolized FF-MAS (substrate of E4), T-MAS (substrate of E5), and lathosterol (substrate of E6), probably to 27-hydroxylated sterols. If the same branches are operative in the liver, CYP7A1 might also have a role. The role of CYP46A1 in the testis is not clear at the moment because we could not detect *Cyp46a1* mRNA expression, but the enzyme might participate in sterol metabolism in other tissues. Dihydrolanosterol (a substrate of E3) was metabolized only by CYP27A1, which is thus the best candidate for E3 in all tissues. Irrespective of the tissue, the early intermediates lanosterol and DHL have less opportunity to escape from the pathway, compared to the intermediates immediately before cholesterol, desmosterol, and 7-DHC, which can be oxidized by CYP11A1, CYP46A1, CYP7A1, and CYP27A1. Sterols from the Bloch and the K-R pathways have the potential to be metabolized further to oxysterols with potentially novel biological activities.

## Materials and Methods

### *In Vivo* Animal Experiments

#### Animals

Forty-two WT and 33 *Crem*^−/−^ male mice of the mixed strain (129S2/SvPasCrlf in C57BL/6JRj) were maintained in a temperature and humidity controlled room under a 12:12 h light: dark cycle (light on at 7:00 am, light off at 7:00 pm) with free access to food (Harlan Teklad 2916) and water. The mice were acclimatised (entrained) to this light/dark cycle for at least one month. *Crem*^−/−^ mice originate from the laboratory of Dr. P. Sassone-Corsi, IGBMC, Strasbourg, and all experiments were performed two years after transfer of animals to Ljubljana. Mice were adults, aged from 56 to 81 days at time of sacrifice. To acquire sufficient information for statistical analysis, identical experimental sampling procedures were repeated for various experimental dates (in April, June, and August). For the two genotype groups, mice were chosen at random over a 24 h sampling procedure.

The experiment was approved by the Veterinary Administration of the Republic of Slovenia (license number 34401-9/2008/4) and was conducted in accordance with the European Convention for the protection of vertebrate animals used for experimental and other scientific purposes (ETS 123), as well as in accordance with National Institutes of Health guidelines for work with laboratory animals.

#### Tissue samples

The mice were sacrificed by cervical dislocation under dim red light, every 4 h over the 24 h period, starting on the second day after their transfer to dark/dark conditions for 24 h. immediately after they were sacrificed, the testes were excised, snap frozen in liquid nitrogen, and stored at −80 °C for later analysis. Alltogether 75 testicular samples from the mixed strain (129S2/SvPasCrlf in C57BL/6JRj) were used for sterol extraction (for further details see references^41^,^42^).

#### RNA extraction

Total RNA was isolated from frozen pulverized testis from five WT and five *Crem*^−/−^ animals according to the manufacturer’s instructions (SigmaAldrich, St. Louis, MO, United States). cDNA preparation and qPCR were performed as described^41^. Reference gene selection and normalization of qPCR data was done as described^43^.

#### Sterol extraction and GC-MS analysis

The frozen testes (50–100 mg per mouse) were dissolved in 2 ml of Folch solution (chloroform/ methanol; 2:1; v/v) under argon and sealed with a Teflon-lined cap. After 24 h at room temperature, 50 μl of the extract for cholesterol and 400 μl of the extract for the cholesterol precursors was transferred into new vials. The internal standards were added as 2 μg of hexadeuterium-labelled cholesterol for the cholesterol analysis and 150 ng of tetradeuterium-labelled lathosterol for the cholesterol precursors. After hydrolysis of the esterified sterols, 3 ml cyclohexane was added, and the upper (organic) phase was transferred to new glass vials; the extraction was repeated once more with a further 3 ml of cyclohexane. The extracts were pooled and evaporated under a stream of argon in heating blocks at 60 °C in preparation for derivatization. The samples were analyzed according to a gas chromatography–mass spectrometry (GC-MS) methodology described previously^44^. Each sample was analyzed for six post-squalene sterols: lanosterol, T-MAS, lathosterol, desmosterol, 7-DHC, and cholesterol. The quantities of the total (i.e. free and esterified) sterols of these mouse testis samples were normalised to the wet tissue weight.

#### Statistical analysis

The R statistical programming language was used for the statistical analysis and the package ggplot2 for the graphical presentations^45^. Comparison of gene expression between the WT and *Crem*^−/−^ lines was performed with t-tests on the logarithmic data. Sterol intermediates were analyzed by ANOVA, adjusted on two extraneous variables (age and experiment date).

### Mathematical Modeling

#### Cholesterol synthesis network model

A mathematical model of cholesterol synthesis was applied^46^ based on object-oriented modeling by combining substance containers (concentrations) and reactions (fluxes) as objects. The basic principles and equations for describing concentrations of substances, reactions that define the fluxes through the metabolic network (enzyme reaction model), and mRNA and enzyme formation and degradation are described in detail in the Supplementary Information. The model was implemented in Dymola 5.3. (Dynasim AB, Lund, Sweden) and simulated with a Petzold integration routine (dassl).

#### Simulation of cholesterol synthesis pathway in the testis of *Crem* ^−/−^ mice: optimizing the model according to the measured genes and metabolites

The initial cholesterol synthesis network model was composed of the pathways confirmed in KEGG, LIPID MAPS, and BioCyc (see Supplementary Fig. S1). The modeling aim was to mechanistically describe the mechanisms that are involved in processes triggered by *Crem*^−/−^. A highly regulated pathway is capable of adapting to wide variety of disruptions, some as heavy as complete inactivation of certain enzymes in the pathway through genetic disorder, while the underlying mechanisms are still not understood due to their high robustness and redundancy. The simplest possible model design was used, where all model values were normalized. The normalized values enabled direct comparison of *Crem*^−/−^ vs. WT ratios obtained from the experiments and reduced the number of the free parameters of the model to minimum.

The model parameters were set by defining arbitrary initial metabolic flux through the network, with 75% of the flux arising from *de novo* cholesterol synthesis and 25% from other resources (dietary cholesterol intake). The initial flux distributions within the network are described in Fig. 3.

#### Model setup

Michaelis-Menten reaction kinetics were used to describe enzyme reactions (hyperbolic plots, fit using non-linear regression). Constant protein biosynthesis and biodegradation with linear kinetics is used in the model. mRNA is also constantly synthesized and degraded with linear kinetics. The flux of protein synthesis depends on the corresponding mRNA concentration and has also linear character. Feedback on gene expression of cholesterogenic enzymes through SREBF2 was not active in the model and mRNA levels were set to values observed during the experiments. All fluxes in the model were considered at steady state at the beginning of the simulation which resulted in a system of linear equations that yielded the values of model parameters. For each reaction, its initial reversibility (ratio of reverse and forward flux) and relative steady-state initial complex concentration were set and, to conserve flux through the network, for each branching point of the network the ratio of dividing initial fluxes was also set. The influence of the reversibility was negligible so all reactions were set to 0.01, and the influence of the steady-state initial complex concentration strongly effects the length of the transient phenomena after the network disturbance. However, it does not affect the new steady-state of the network and therefore it was set to 0.01 (1% of the initial normalized value of the substrate or enzyme) to shorten the transient phenomena (small values of steady-state complex concentration indicate fast reactions and thus faster transient phenomena). The division of fluxes at branching points can significantly influence the new steady-state of the network therefore they must be carefully chosen. Using literature data for mice testis we were able to set the flux divisions ratios for the textbook cholesterol biosynthesis model.

#### Simulation setup

Eight hypotheses were tested with the model.

0. The textbook model is sufficient to describe the *Crem*^−/−^ situation in comparison with the WT situation and the only influence on enzyme activity is through gene expression modulation.

1. The textbook model correctly describes the *Crem*^−/−^ - WT relations, enzyme activity is regulated only through gene expression except for HMGCR where known degradation of HMGCR through high levels of lanosterol can produce lower activity than expected by just observing *Hmgcr* expression.

2. The textbook model correctly describes the *Crem*^−/−^ - WT relationship; however, the enzyme activities are not solely regulated by gene expression but may be affected by other factors.

3. The textbook model correctly describes the *Crem*^−/−^ - WT; however, flux distributions through the network are not correct.

4. The textbook model is too simple to describe the *Crem*^−/−^ - WT relations and DHL is eliminated from the pathway via an alternative route. Enzyme activities are regulated through gene expression as well as protein degradation mechanisms.

5. The textbook model is too simple to describe the *Crem*^−/−^ - WT relations, and DHL and lathosterol are eliminated from the pathway via alternative routes. Enzyme activities are regulated through gene expression as well as protein degradation mechanisms.

6. The textbook model is too simple to describe the *Crem*^−/−^ - WT relations and DHL, lathosterol, and FF-MAS are eliminated from the pathway via alternative routes. Enzyme activities are regulated through gene expression as well as protein degradation mechanisms.

7. The textbook model is too simple to describe the *Crem*^−/−^ - WT relations and DHL, lathosterol, FF-MAS, and T-MAS are eliminated from the pathway via alternative routes. Enzyme activities are regulated through gene expression as well as protein degradation mechanisms.

Optimal enzyme activities and flux distribution ratios were estimated using criterion minimization. The criterion was the sum of squared differences between measured and simulated relative changes in steady-state concentrations of metabolites: lanosterol, T-MAS, lathosterol, desmosterol, and cholesterol. The initial steady-state for all model concentrations was 1; therefore, the steady-state values after perturbation were already relative changes of concentrations. Simulation end time was selected well beyond transient phenomena and was monitored throughout the optimization process through concentration derivative at the simulation end. Nelder-Mead optimization routine was used. Multiple local minima were expected since in most case the number of parameters to be optimized was higher than the number of metabolite concentrations to be fitted. This creates a situation where degrees of freedom are usually higher than necessary for solving the problem which results in several combinations that produce the same result. To explore the multiple solutions problem, the optimization was started from several starting points. One initial point was original enzyme activity, the next initial point was original enzyme activity except for HMGCR where reduced activity was used, and the final initial point was optimal estimated enzyme activity of the final model. The enzyme activity was modulated by tuning the enzyme elimination coefficient.

### Enzyme Activity Experiments

#### Codon optimization and construction of expression vectors

On-line software (Integrated DNA Technologies, Coralville, IA; http://www.idtdna.com/CodonOpt) was used for codon optimization for human CYP11A1. A cDNA containing an optimized coding sequence (Fig. S22) and a C-terminal (His)_6_ tag was synthesized and ligated into a pCW expression vector (using *Nde*I and *Hind*III restriction sites) by Genewiz (South Plainfield, NJ, United States).

A tricistronic plasmid construct containing human CYP27A1, adrenodoxin (ADX), and adrenodoxin reductase (ADR)^47^ was used as a template for PCR to amplify the cDNA of CYP27A1. The 5′ primer for CYP27A1 insertion into the pCW was designed to introduce the *Nde*I restriction site within the initiation codon ATG (Met). The sequence of the 5′ primer was CATATGGCTCTTCCATCTGATAAA. The 3′ primer was template-specific but with a C-terminal (His)_6_-tag, stop codon (TAA), and *Xba*I restriction site. The sequence of the 3′ primer was GCAGTTCCTGCAGAGACAGTGC CACCATCACCATCACCATTAATCTAGA. The PCR products were digested and ligated into the pCW vector, and the sequence of the insert confirmed by nucleotide sequence analysis.

#### Enzyme expression, purification, and activity assays

Expression and purification of CYP7A1 and CYP46A1 were described earlier^20^,^48^. Recombinant rat NADPH-cytochrome P450 reductase (CPR) was expressed in *Escherichia coli* and purified as described^49^.

Detailed protocols for expression and purification of CYP11A1 and CYP27A1 with ferrous-carbon monoxide vs. ferrous difference spectra^50^ (Fig. S23) as well as ADX and ADR are provided in the Supplementary Information.

Activity assays were carried out in a 0.5 ml reaction volume containing the indicated sterol (7-DHC, cholesterol, lanosterol, DHL, FF-MAS, T-MAS, zymosterol, 7-dehydrodesmosterol, zymostenol, lathosterol, or desmosterol), the CYP enzyme and its redox partner: CPR for CYP46A1 and CYP7A1 or ADX and ADR for CYP27A1 and CYP11A1. Experimental details are described in the Supplementary Information.

#### Sterol separation and identification

The various products obtained from enzymatic assays with all CYPs were separated and identified using LC-MS and confirmed either by comparing with authentic standards (7*α*-hydroxycholesterol, pregnenolone, 27-hydroxycholesterol, and 24-hydroxycholesterol), or GC-MS fragmentation analysis, or NMR. Experimental details are provided in the Supplementary Information.

## Additional Information

**How to cite this article**: Ačimovič, J. *et al*. Cytochrome P450 metabolism of the post-lanosterol intermediates explains enigmas of cholesterol synthesis. *Sci. Rep.* **6**, 28462; doi: 10.1038/srep28462 (2016).

## Supplementary Material

Supplementary Information

## Figures and Tables

**Figure 5:**
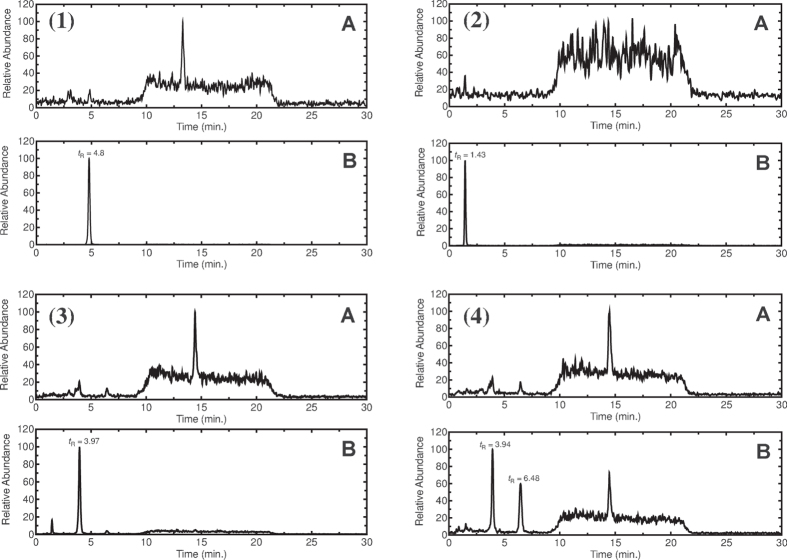
LC-MS profiles of desmosterol incubation with different CYP enzymes. (1) CYP7A1, (2) CYP11A1, (3) CYP27A1, and (4) CYP46A1. An APCI^+^ ionization mode was used, and *m*/*z* 383 (for hydroxy and epoxy products, with loss of H_2_O to give *m*/*z* 383 [MH-18]^+^) was monitored in each case except CYP11A1 where it was monitored at *m*/*z* 299 (for pregnenolone, with loss of H_2_O to give *m*/*z* 299 [MH-18]^+^). A, assay without enzyme. B, assay with enzyme.

**Table 1:** Experimental and simulated results.

mRNA		Cholesterol synthesis intermediates		
Gene	Change from WT Mean (95% CI)	Sterol	measured Mean (95% CI)	simulated
*Hmgcr*	1.65 (1.38–1.91)			
E1				
*Fdft1*	3.09 (2.48–3.71)			
E2				
*Sqle*	1.42 (1.06–1.77)			
*Lss*	1.14 (0.65–1.63)*			
*Cyp51*	0.39 (0.17–0.68)	Lanosterol	3.28 (2.59–4.16)	3.28
*Dhcr24*	1.21 (0.94–1.48)*			
E3				
E4				
*Tm7sf2*	0.87 (0.55–1.18)*			
E5				
*Sc4mol*	1.30 (0.61–1.99)*	T-MAS	0.26 (0.19–0.36)	0.26
*Nsdhl*	0.87 (0.50–1.24)*			
*Hsd17b7*	1.55 (1.06–1.34)			
*Ebp*	1.81 (1.47–2.15)	Lathosterol	0.53 (0.46–0.61)	0.53
E6				
*Sc5d*	0.90 (0.70–1.11)*			
*Dhcr7*	0.29 (0.05–0.54)	7-DHL	0.86 (0.78–0.94)	0.86
*Cyp11a1*	1.74 (1.28–2.21)			
		Desmosterol	0.88 (0.78–0.99)	0.88
		Cholesterol	1.15 (1.08–1.23)	1.15

Experimental results (with corresponding 95% confidence intervals, real measurements)) in comparison with simulated data (sim) for expression of several genes and cholesterol synthetic sterol intermediates of the best fit model (Experiment Seven). Values represent the ratio of *Crem*^−/−^ versus WT.

^*^Statistically non-significant; CI, confidence interval.

**Table 2:** Summary of enzymatic assays with CYPs and various sterols.

CYP	Sterols	Products	Ref.
CYP7A1	Zymostenol	^*^n.d.	This work
	Lathosterol	7-Ketocholestanol	36
		Cholestanol-7*α*,8*α* -epoxide	
	7-DHC	7-Ketocholesterol	
	Desmosterol	7α-Hydroxydesmosterol	This work
	Cholesterol	7α-Hydroxycholesterol	
CYP11A1	Zymostenol	^#^n.r.	
	Lathosterol	^#^n.r.	
	7-DHC	7-Dehydropregnenolone	
	Desmosterol	Pregnenolone	
	Cholesterol	Pregnenolone	
CYP27A1	Zymostenol	25-Hydroxyzymostenol	
		27-Hydroxyzymostenol	
	Lathosterol	25-Hydroxylathosterol	
		27-Hydroxylathosterol	
	7-DHC	25-Hydroxy-7-dehydrocholesterol	
		27-Hydroxy-7-dehydrocholesterol	
	Desmosterol	27*S*,25-Epoxycholesterol	
	Cholesterol	24*S*-Hydroxycholesterol	
CYP46A1	Zymostenol	24-Hydroxyzymostenol	
		25-Hydroxyzymostenol	
	Lathosterol	24-Hydroxylathosterol	
		25-Hydroxylathosterol	
	7-DHC	24-Hydroxy-7-dehydrocholesterol	20
		25-Hydroxy-7-dehydrocholesterol	
	Desmosterol	24*S*,25-Epoxycholesterol	
		27-Hydroxydesmosterol	
	Cholesterol	24*S*-Hydroxycholesterol	
*n.d.	could not determine (reaction detected, insufficient separation)		
^#^n.r.	no reaction detected		

Reactions with cholesterol served as positive control.

**Table 3:** Metabolism of sterol intermediates from the Bloch and Kandutch-Russell (K-R) pathway by CYP enzymes that are known to metabolize cholesterol.

Bloch pathway	CYP7A1	CYP11A1	CYP27A1	CYP46A1
Lanosterol			15%	
FF-MAS	1%		5%	5%
T-MAS	5%		50%	1%
Zymosterol	65%		75%	4%
7-Dehydrodesmosterol	25%		83%	9%
Desmosterol	+	+^a^	+	+^b^
K-R pathway	CYP7A1	CYP11A1	CYP27A1	CYP46A1
Dihydrolanosterol			16%	
Zymostenol	45%		+	+
Lathosterol	+^c^		+	+
7-DHC	+^d^	+	+	+^e^

For percent conversion, each CYP was present (in a reconstituted system) at 1 μM and the substrate concentration was 10–25 μM. The reaction time was 15 min. Shown is the % conversion of substrates to products that were not identified. The identified products (indicated with a “+” sign) are presented in Table 2.

^*a*^The *k*_cat_ for formation of pregnenolone was 4.6 ± 0.15 min^−1^, *K*_m_ 1.2 ± 0.36 μM (this work).

^*b*^The *k*_cat_ for formation of 24*S*,25-epoxycholesterol was 0.033 ± 0.001 min^−1^, *K*_m_ 2.17 ± 0.28 μM^20^. The *k*_cat_ for formation of 27-hydroxydesmosterol was ~0.044 min^−1 ^20.

^*c*^The *k*_cat_ for formation of 7-ketocholestenol was 3.7 ± 0.2 min^−1^, *K*_m_ 1.8 ± 0.3 μM, and the *k*_cat_ for formation of 7*α*,8*α*-epoxycholestenol was 7.1 ± 0.4 min^−1^, *K*_m_ 2.1 ± 0.3 μM^36^.

^*d*^The *k*_cat_ for formation of 7-ketocholesterol was 2.2 ± 0.1 min^−1^, *K*_m_ 1.1 ± 0.1 μM^36^.

^*e*^The *k*_cat_ for formation of 24-hydroxy-7-dehydrocholesterol was 0.024 ± 0.001 min^−1^, *K*_m_ 0.24 ± 0.008 μM and the *k*_cat_ for formation of 25-hydroxy-7-dehydrocholesterol was ~0.11 min^−1 ^20.
