# Supplementary material for: Cytochrome P450 metabolism of the post-lanosterol intermediates explains enigmas of cholesterol synthesis
Source: Sci Rep. 2016 Jun 23;6:28462. doi: 10.1038/srep28462 (PMC4917857; doi:10.1038/srep28462)
Supplement: Supplementary Information [file srep28462-s1.pdf]

Supplementary Information

Cytochrome P450 metabolism of the post-lanosterol intermediates explains enigmas of cholesterol synthesis

Jure Ačimovič[1,2, &], Sandeep Goyal[3,6, &], Rok Košir[1,2], Marko Goličnik[2], Martina Perše[4], Ales Belič[5], Žiga Urlep[1,2], F. Peter Guengerich[3,#], Damjana Rozman[1,2,#,*]

[1]Center for Functional Genomics and Bio-Chips, Faculty of Medicine, University of Ljubljana, Zaloška 4, SI-1000 Ljubljana, Slovenia

[2]Institute of Biochemistry, Faculty of Medicine, University of Ljubljana, Vrazov trg 2, SI-1000 Ljubljana, Slovenia

[3]Department of Biochemistry, Vanderbilt University School of Medicine, Nashville, TN 37232-0146, United States

[4]Medical Experimental Centre, Institute of Pathology, Faculty of Medicine, University of Ljubljana, Zaloška 4, SI-1000 Ljubljana, Slovenia

[5] Faculty of Electrical Engineering, University of Ljubljana, Slovenia

[6]Current Affiliation: Department of Chemistry and Biochemistry, University of Arizona, Tucson, AZ 85721, United States

[&]The authors contributed equally to this work

[#]The P.I.s contributed equally to this work

[*]Corresponding author
DR: damjana.rozman@mf.uni-lj.si

Abbreviations

K-R/Kandutsch-Russell, DHL/24,25-dihydrolanosterol, 7-DHC/7-dehydrocholesterol, T-MAS/testis meiosis-activating sterol, CYP/cytochrome P450, CREM/cAMP responsive element modulator, WT/wild-type, FF-MAS/follicular-fluid meiosis-activating sterol, GC-MS/gas chromatography-mass spectrometry, LC-MS/liquid chromatography-mass spectrometry, HSQC/heteronuclear single-quantum correlation (NMR) spectroscopy, COSY/correlation (NMR) spectroscopy, SREBF/sterol regulatory element binding transcription factor, RORγ/RAR-related orphan receptor gamma, ADX/Adrenodoxin, ADR/NADPH-adrenodoxin reductase, CPR/Recombinant rat NADPH-cytochrome P450 reductase

<u>Enzyme (gene) abbreviations are according to Unigene:</u>

HMGCR(Hmgcr)/3-hydroxy-3-methylglutaryl-CoA-reductase, FDFT1(Fdft1)/farnesyl-diphosphate-farnesyltransferase-1, SQLE(Sqle)/squalene-epoxidase, LSS(Lss)/lanosterol-synthase, CYP51(Cyp51)/lanosterol-14α-demethylase, DHCR24(Dhcr24)/sterol-Δ^{24}-reductase, DHCR14(Tm7sf2)/sterol-Δ^{14}-reductase, Sc4mol(Sc4mol)/sterol-C4 methyl-oxidase, Nsdhl(Nsdhl)/3*β*-hydroxy-Δ^{5}-steroid-dehydrogenase, HSD17B7(Hsd17b7)/3β-keto-reductase, Ebp(Ebp)/sterol-$\Delta^{8,7}$-isomerase, Sc5d(Sc5d)/sterol-C5-desaturase, Dhcr7(Dhcr7)/sterol-Δ^{7}-reductase, CYP11A1(Cyp11a1)/cytochrome P450-family-11-subfamily-A-polypeptide-1, CYP27A1(Cyp27a1)/cytochrome P450, family 27, subfamily A, polypeptide 1, CYP7A1(Cyp7a1)/cytochrome P450, family 7, subfamily A, polypeptide 1, CYP46A1(Cyp46a1)/cytochrome P450, family 46, subfamily A, polypeptide 1

Supplementary Figures

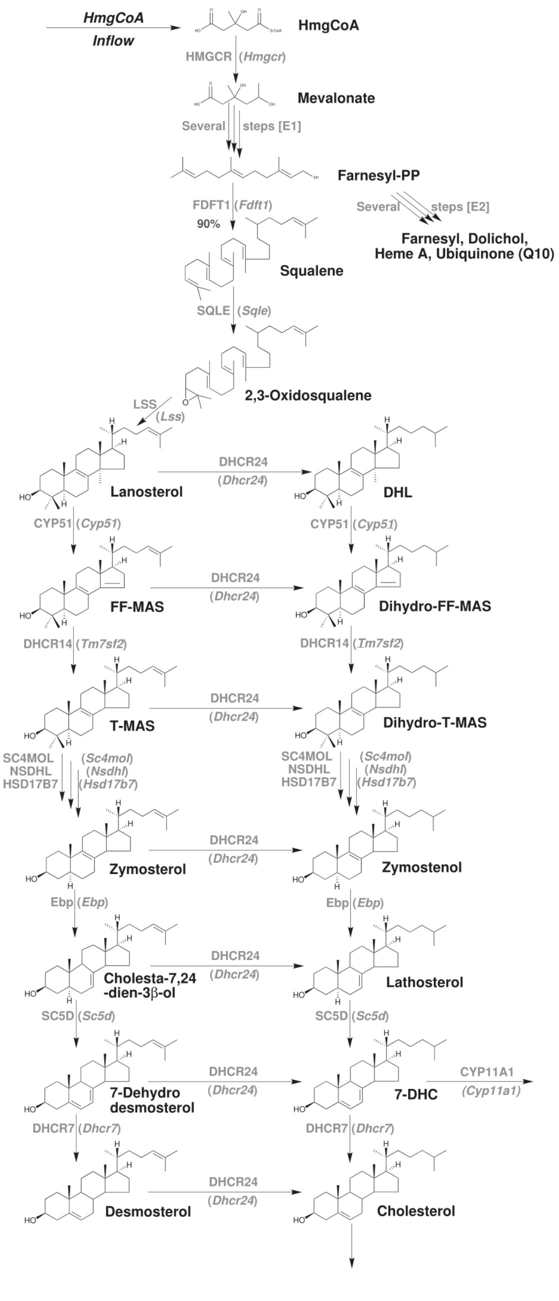

Figure S1. The textbook cholesterol synthesis pathway

Genes (enzymes) in gray and metabolites in black. Enzyme (gene) abbreviations are according to Unigene (see List of Abbreviations). DHL, 24,25-dihydrolanosterol; FF-MAS, follicular-fluid meiosis-activating sterol; T-MAS, testis meiosis-activating sterol; 7-DHC, 7-dehydrocholesterol.

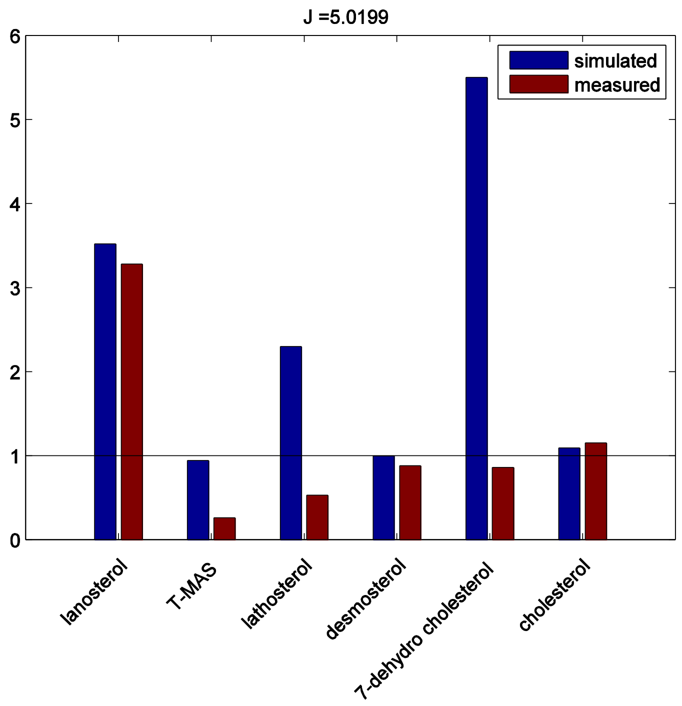

Figure S2. Relative changes of metabolite concentrations of *Crem*$^{-/-}$ vs. WT mice using unmodified textbook model (model 0). J denotes achieved criterion value.

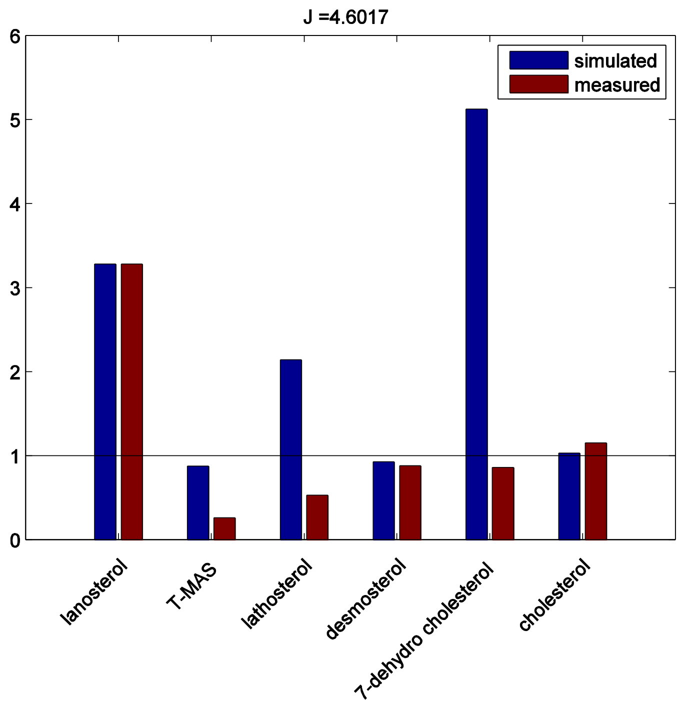

Figure S3. Relative changes of metabolite concentrations *Crem*$^{-/-}$ vs. WT using unmodified textbook model with adapted HMGCR activity (model 1). J denotes achieved criterion value.

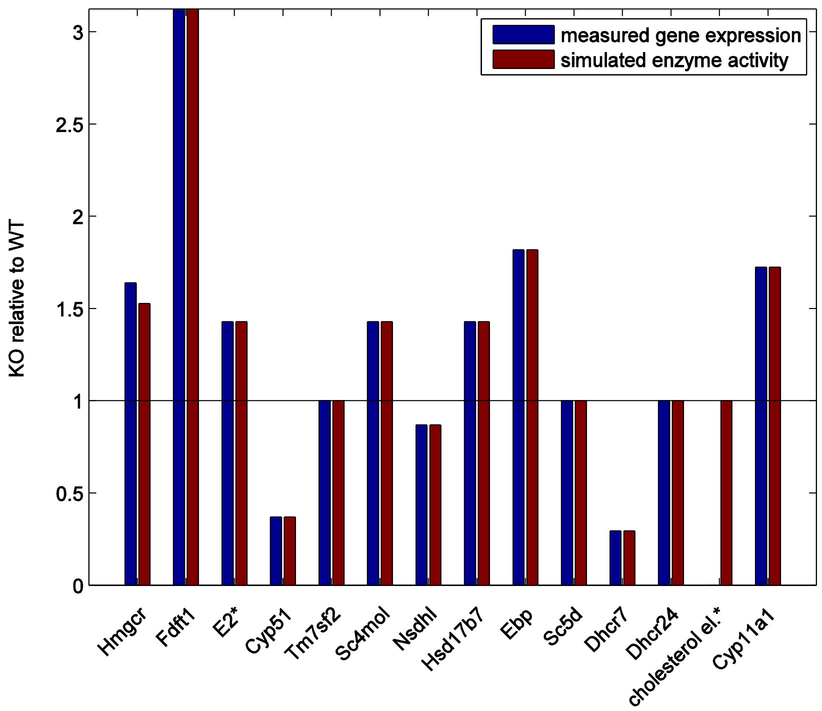

Figure S4. Measured relative gene expressions and simulated enzyme activities (model 1). * - many real early part cholesterol biosynthesis genes joined in one hypothetical gene. Where no gene expression bar is visible, the expression was not measured.

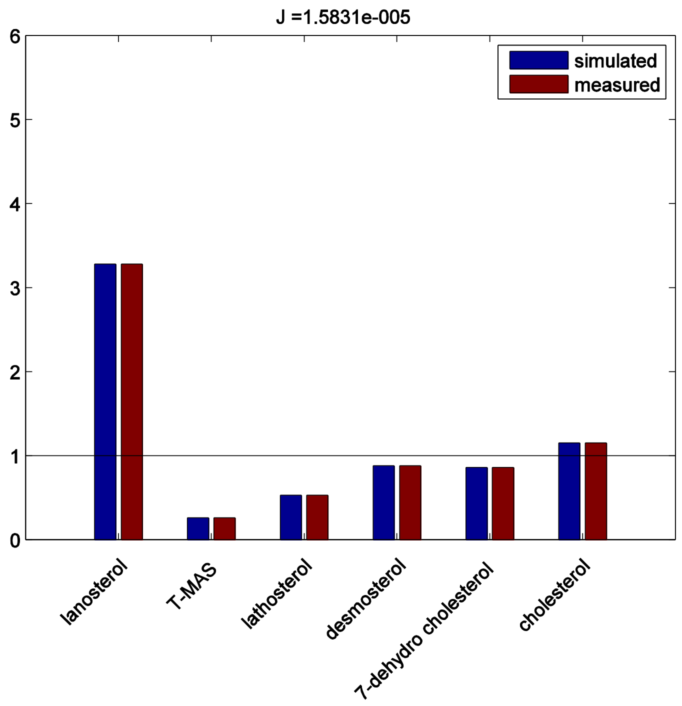

Figure S5. Relative changes of metabolite concentrations *Crem*$^{-/-}$ vs. WT using unmodified textbook model (model 2) with *unlimited* enzyme activities optimisation. J denotes achieved criterion value.

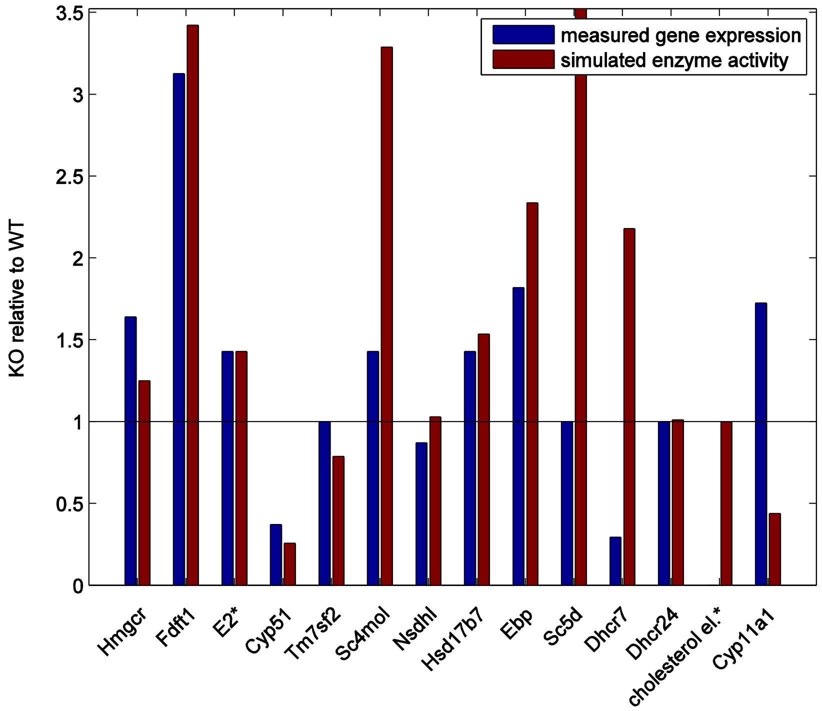

Figure S6. Measured relative gene expressions and simulated enzyme activities for *unlimited* enzyme activities adaptation (model 2). * - many real early part cholesterol biosynthesis genes joined in one hypothetical gene. Where no gene expression bar is visible, the expression was not measured.

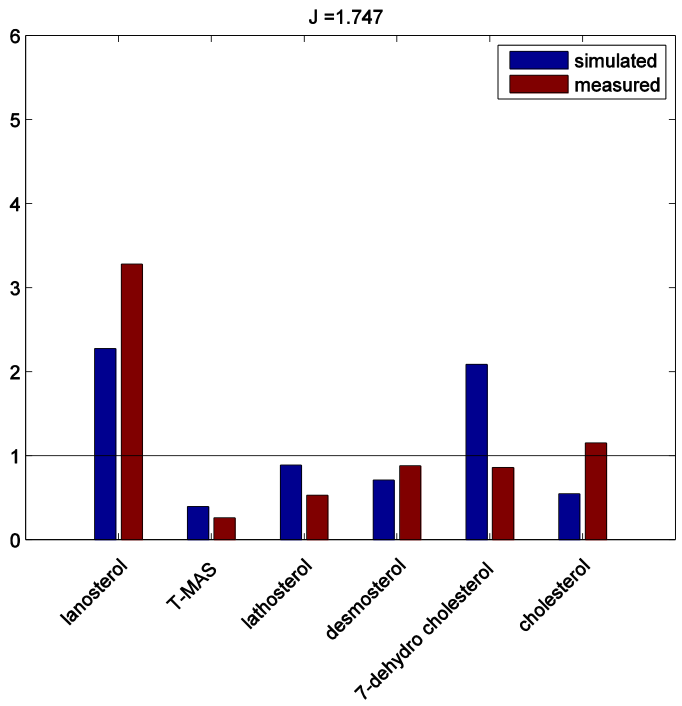

Figure S7. Relative changes of metabolite concentrations *Crem*$^{-/-}$ vs. WT using unmodified textbook model (model 2) with *limited* enzyme activities optimisation. J denotes achieved criterion value.

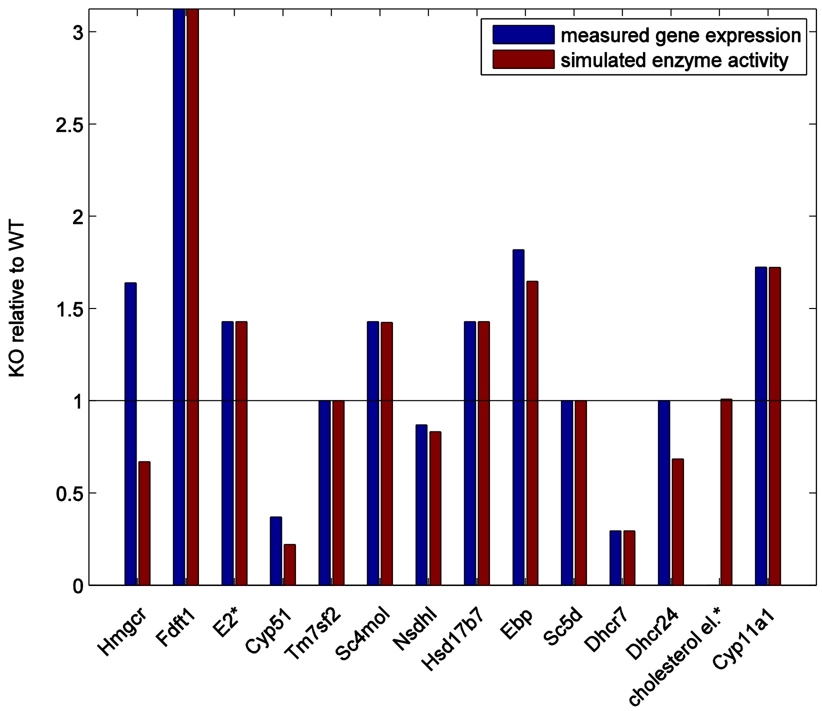

Figure S8. Measured relative gene expressions and simulated enzyme activities for *limited* enzyme activities adaptation (model 2). * - many real early part cholesterol biosynthesis genes joined in one hypothetical gene. Where no gene expression bar is visible, the expression was not measured.

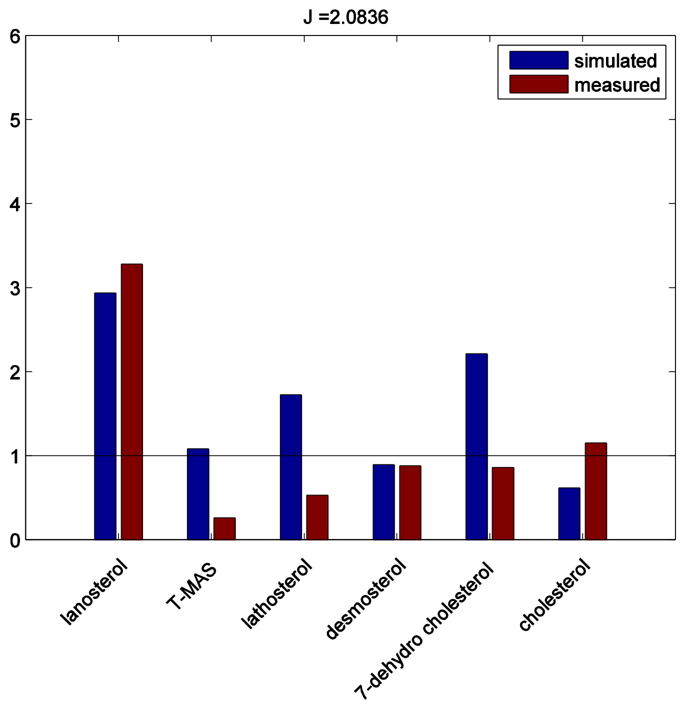

Figure S9. Relative changes of metabolite concentrations *Crem*$^{-/-}$ vs. WT using unmodified textbook model with adapted flux distribution through the model (model 3). J denotes achieved criterion value.

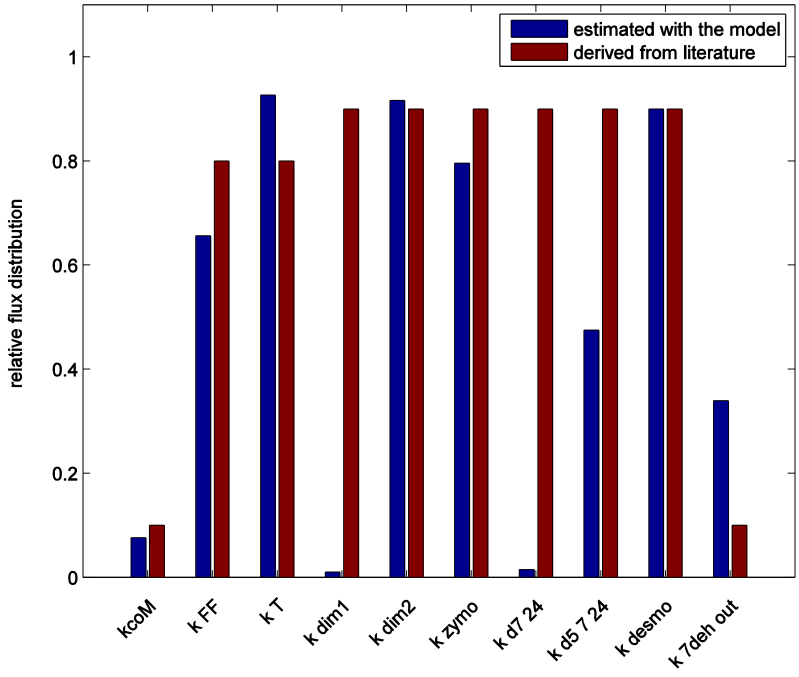

Figure S10. Distribution of fluxes through the model 3: kcoM – share of flux towards farnesyl, k FF – share of flux toward FF-MAS, k T – share of flux toward T-MAS, k dim1 – combined Bloch pathway flux towards zymosterol, k dim2 – combined Bloch pathway flux towards zymosterol), k zymo – share of flux toward zymosterol, k d7 24 – share of flux towards Δ7,24 cholestadienol, k d5 7 24 – share of flux towards Δ5,7,24 cholestatrienol, k desmo – share of flux toward desmosterol, k 7deh out – share of flux towards vitamin D.

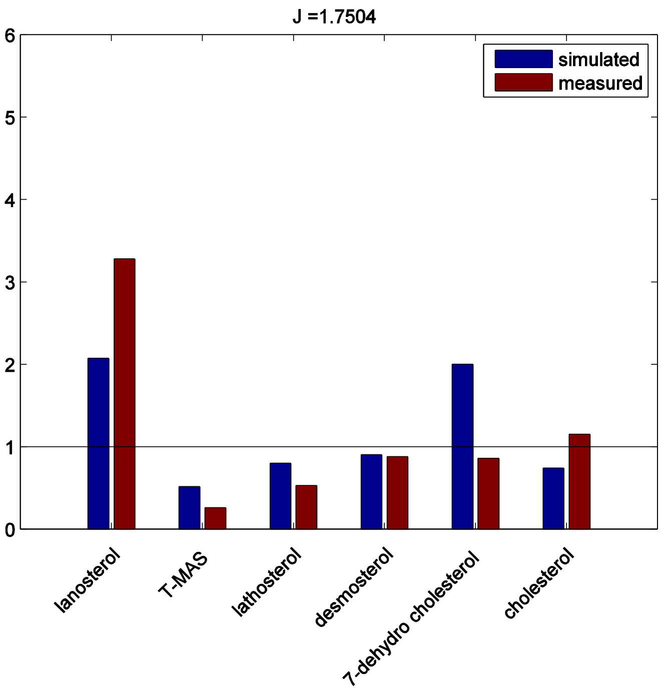

Figure S11. Relative changes of metabolite concentrations *Crem*$^{-/-}$ vs. WT using textbook model with added elimination for 24,25-dihydrolanosterol (model 4) with limited enzyme activities optimisation. J denotes achieved criterion value.

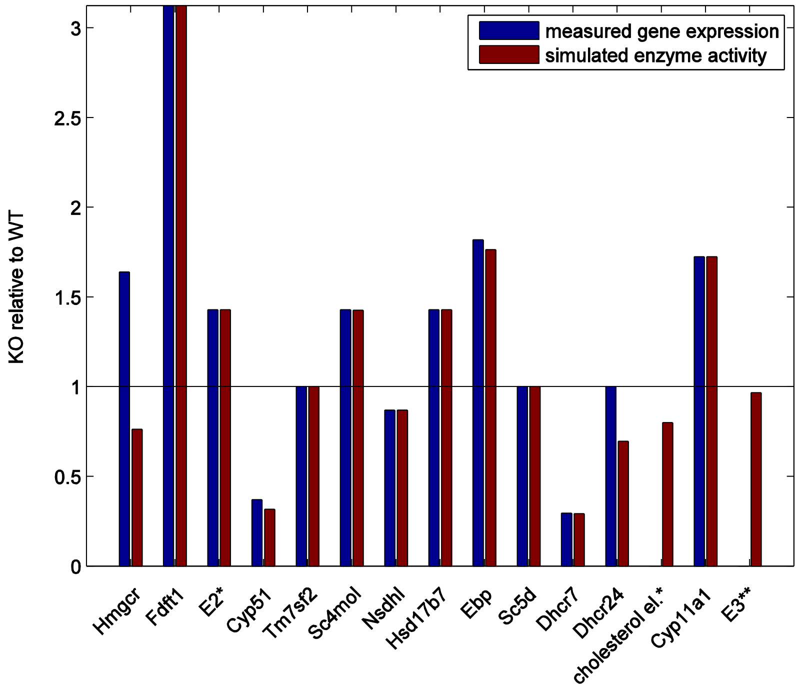

Figure S12. Measured relative gene expressions and simulated enzyme activities for limited enzyme activities adaptation (model 4). * - many real early part cholesterol biosynthesis genes joined in one hypothetical gene, ** - hypothetical genes. Where no gene expression bar is visible, the expression was not measured.

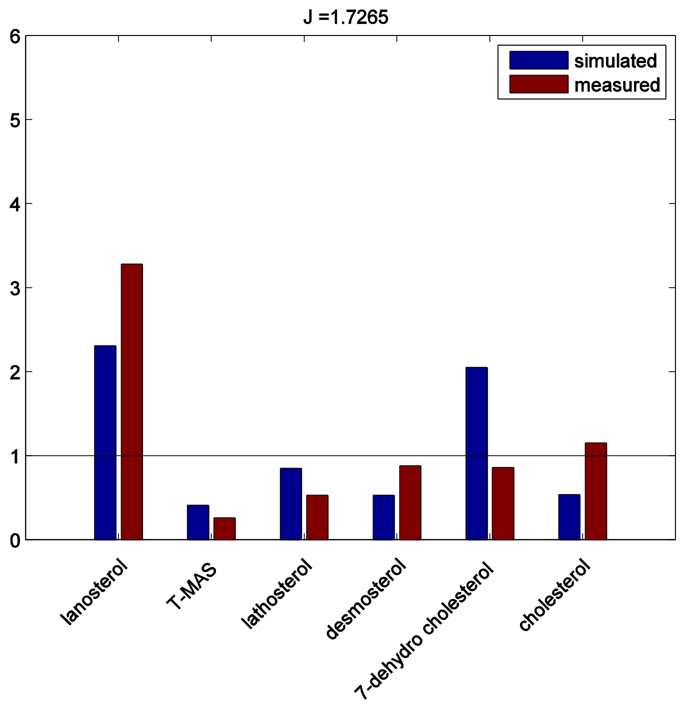

Figure S13. Relative changes of metabolite concentrations *Crem*$^{-/-}$ vs. WT using textbook model with added elimination for 24,25-dihydrolanosterol and lathosterol (model 5) with limited enzyme activities optimization. J denotes achieved criterion value.

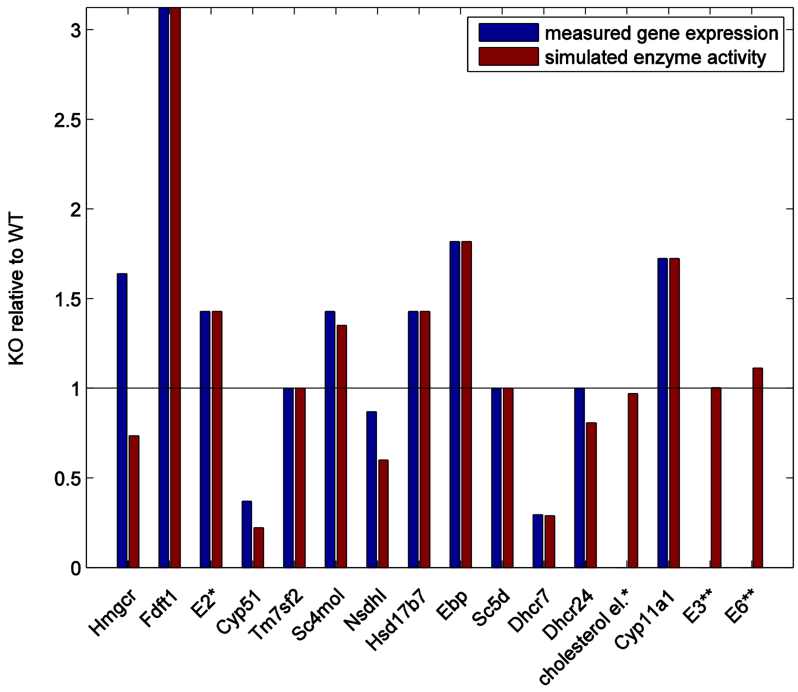

Figure S14. Measured relative gene expressions and simulated enzyme activities for limited enzyme activities adaptation (model 5). * - many real early part cholesterol biosynthesis genes joined in one hypothetical gene, ** - hypothetical genes. Where no gene expression bar is visible, the expression was not measured.

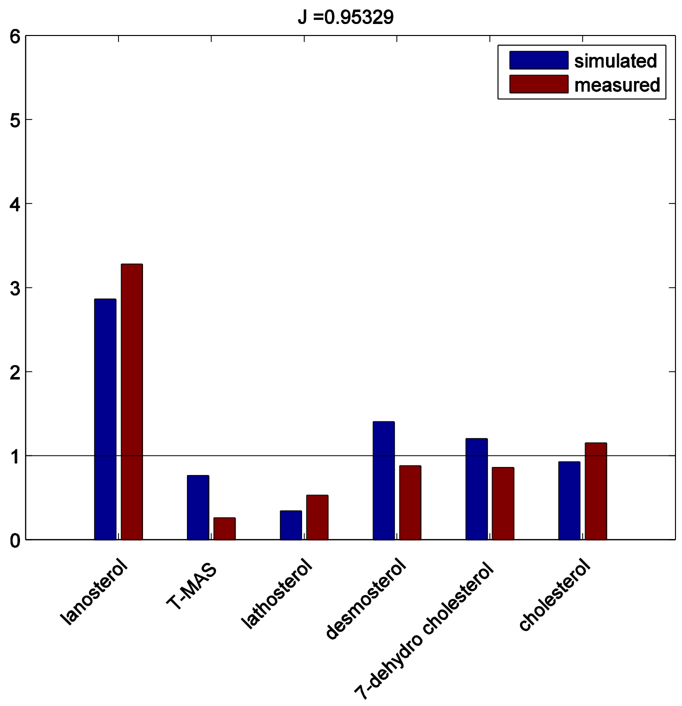

Figure S15. Relative changes of metabolite concentrations *Crem*$^{-/-}$ vs. WT using textbook model with added elimination for dehydro lanosterol, lathosterol and FF-MAS (model 6) with limited enzyme activities optimization. J denotes achieved criterion value.

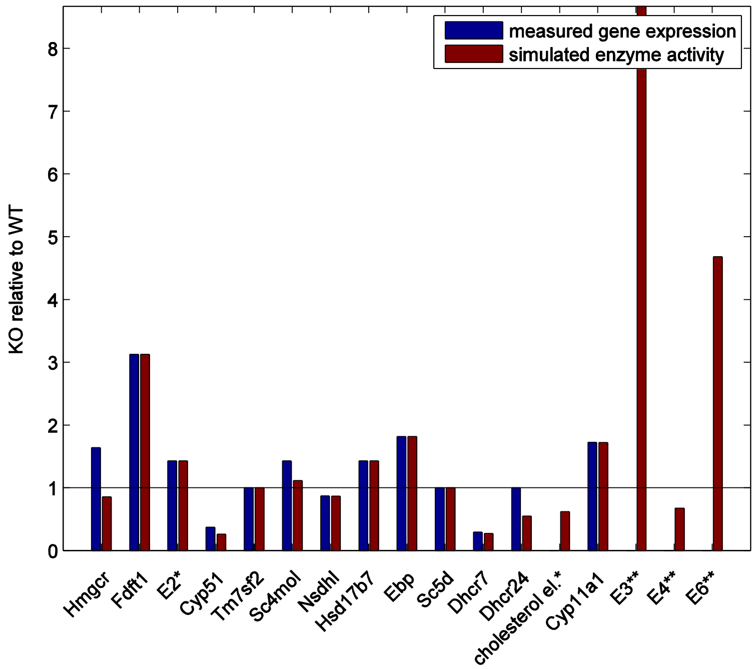

Figure S16. Measured relative gene expressions and simulated enzyme activities for limited enzyme activities adaptation (model 6). * - many real early part cholesterol biosynthesis genes joined in one hypothetical gene, ** - hypothetical genes. Where no gene expression bar is visible, the expression was not measured.

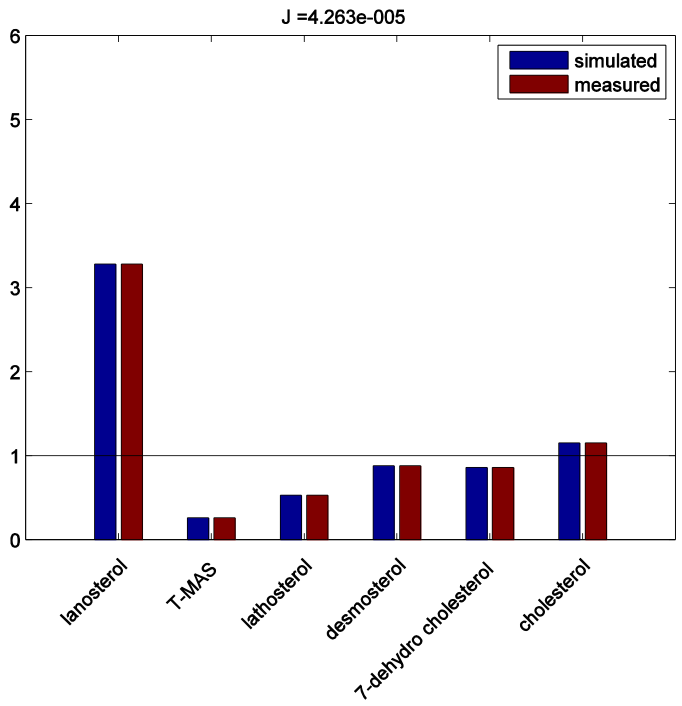

Figure S17. Relative changes of metabolite concentrations *Crem*$^{-/-}$ vs. WT using textbook model with added elimination for DHL, lathosterol, FF-MAS, and T-MAS (model 7 – final model) with limited enzyme activities optimization. J denotes achieved criterion value.

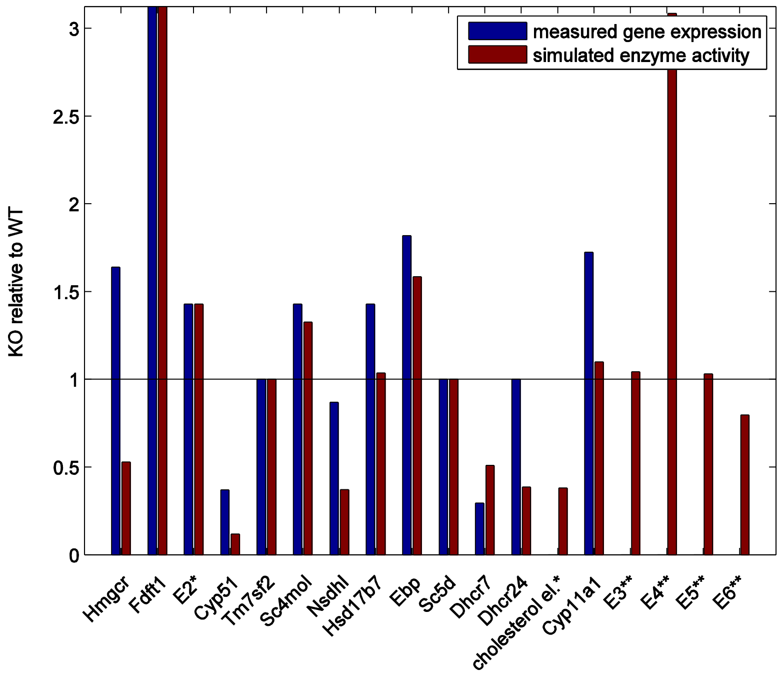

Figure S18. Measured relative gene expressions and simulated enzyme activities for limited enzyme activities adaptation (model 7 - final model). * - many real early part cholesterol biosynthesis genes joined in one hypothetical gene, ** - hypothetical genes. Where no gene expression bar is visible, the expression was not measured.

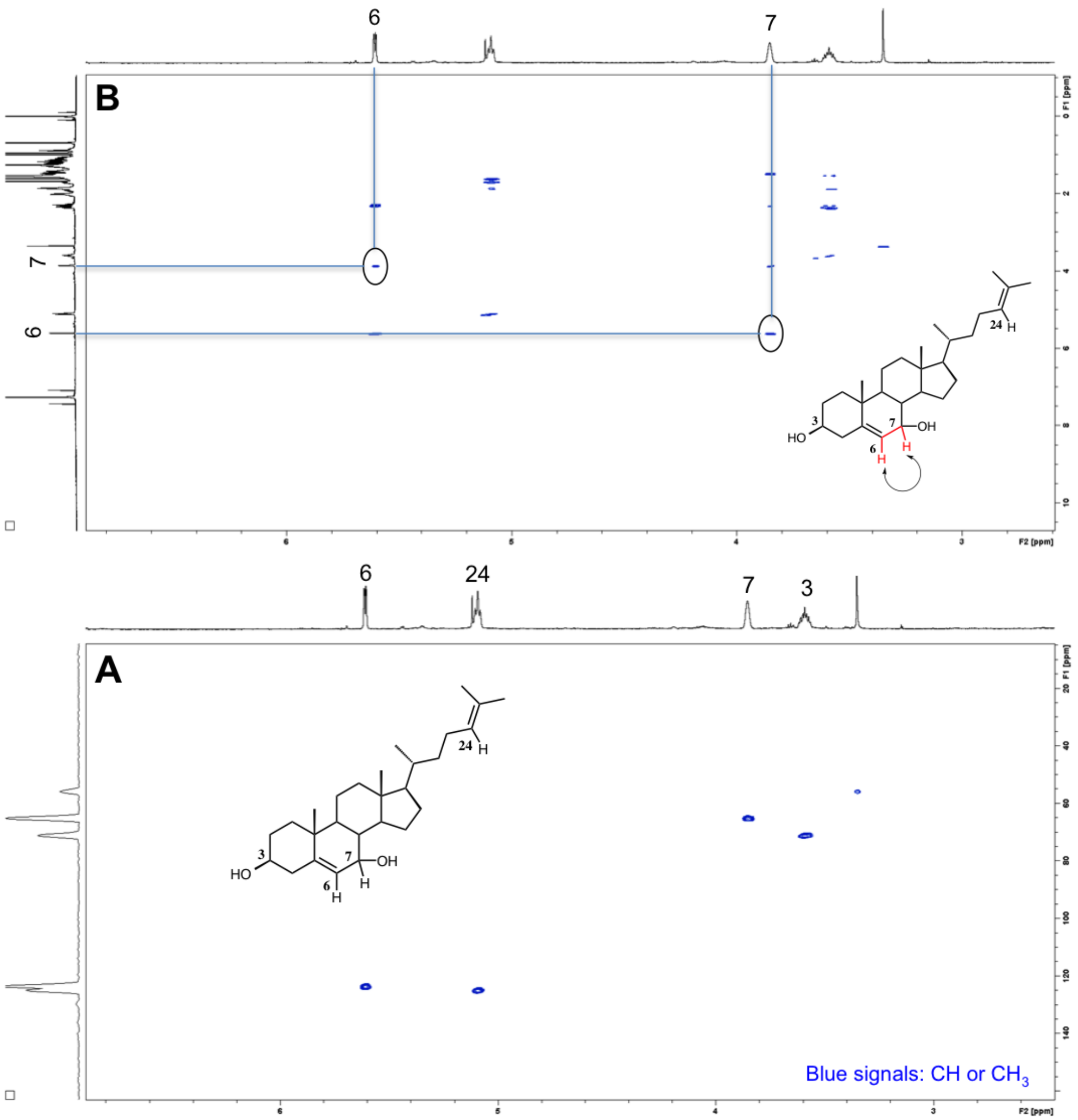

Figure S19. 2D NMR spectra of the product obtained from oxidation of desmosterol by CYP7A1. (A) HSQC (B) COSY. The COSY analysis (B) shows the coupling of vinyl (H-6) and carbinol (H-7) protons, confirming the assignment as 7-hydroxydesmosterol.

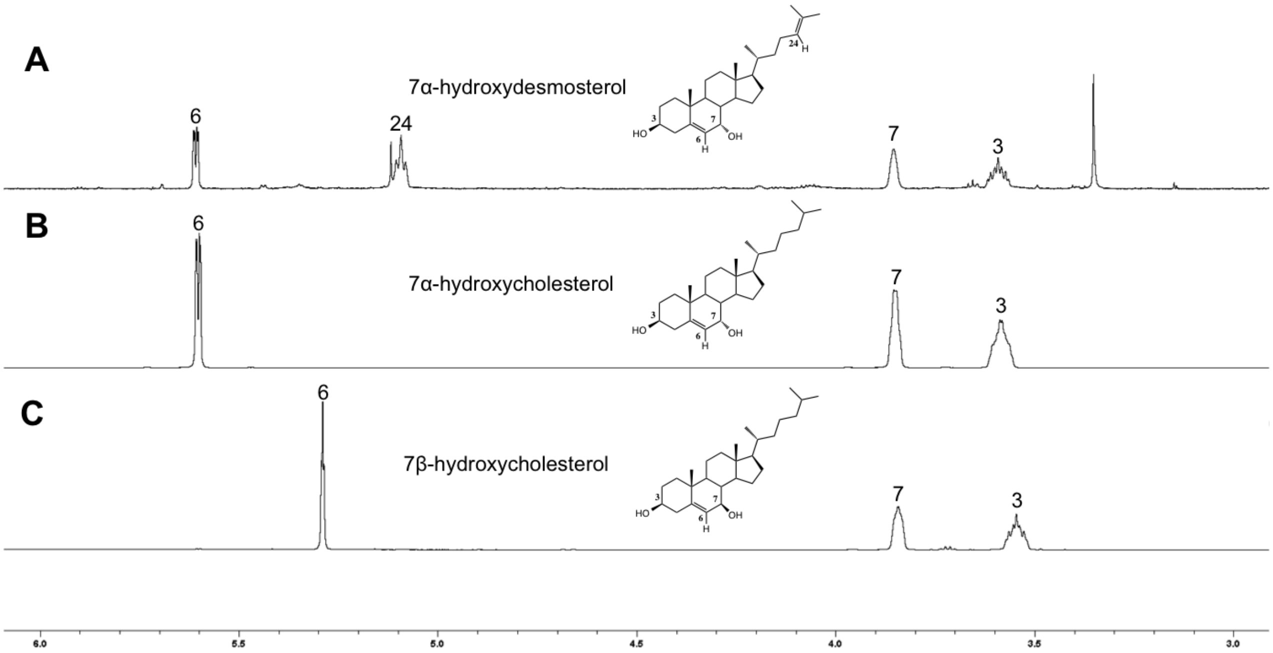

Figure S20. Comparison of ^{1}H NMR spectra of (A) product obtained from oxidation of desmosterol by CYP7A1 (7*α*-hydroxydesmosterol product) and of standard (B) 7*α*-hydroxycholesterol and (C) 7*β*-hydroxycholesterol. The chemical shift of the H-6 proton was used in making the (7) *α* assignment.

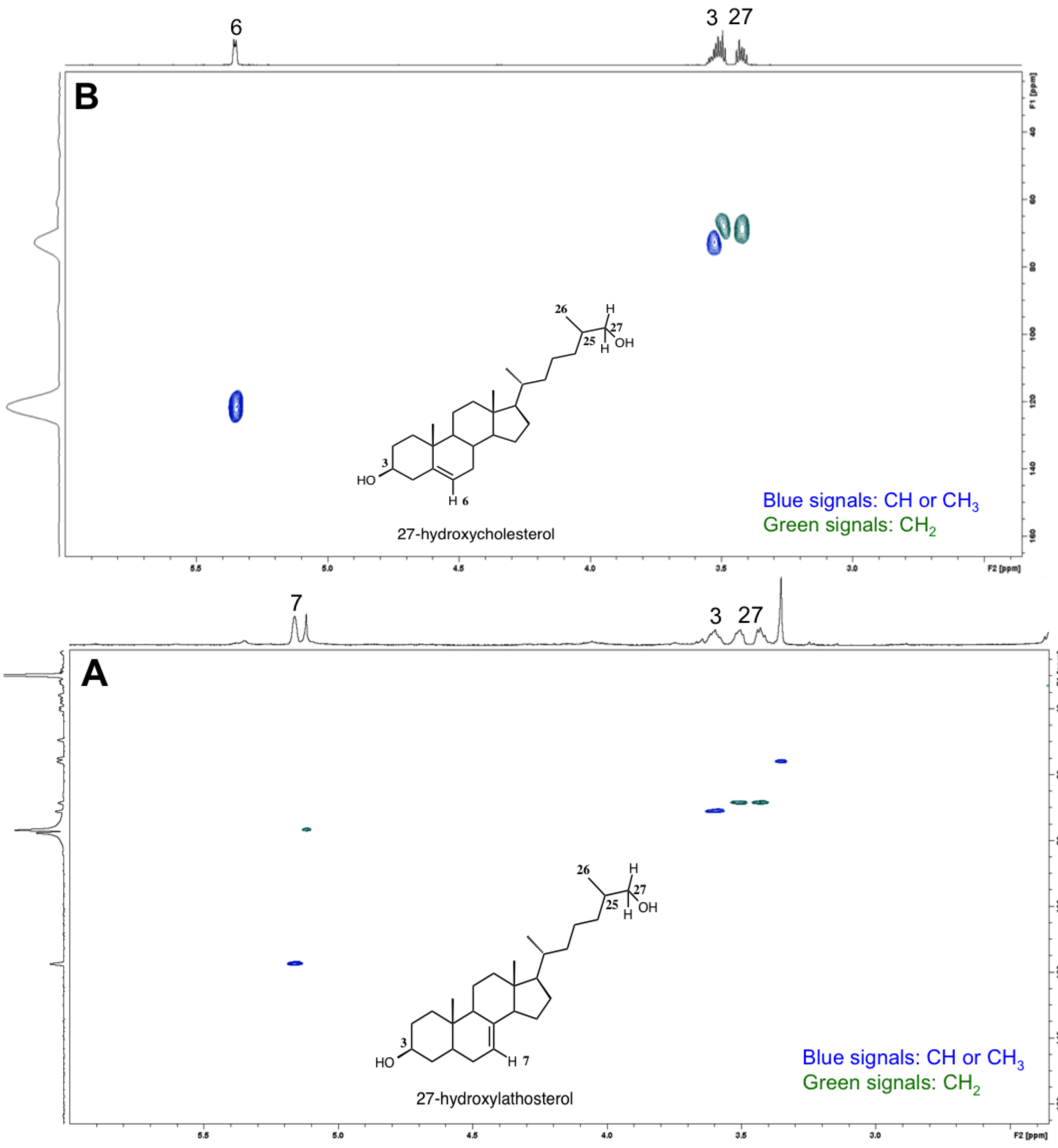

Figure S21. HSQC NMR spectra of (A) product obtained from CYP27A1 and lathosterol and of (B) Standard 27-hydroxycholesterol. The assignment of 27-substitution was based on the comparison of chemical shifts with 27-hydroxycholesterol.

Figure S22. Optimized codon sequence used for synthesis of CYP11A1 cDNA.

ATGGCAAGCACGCGCAGCCCGCGTCCGTTTAACGAAATTCCGTCTCCAGGCGACAACGGA

M A S T R S P R P F N E I P S P G D N G
TGGCTGAATCTCTATCACTTTTGGCGTGAAACGGGCACTCATAAAGTTCACCTGCATCAC

W L N L Y H F W R E T G T H K V H L H H
GTCCAGAACTTCCAGAAGTACGGCCCTATCTACCGCGAAAAGCTGGGGAATGTTGAAAGT

V Q N F Q K Y G P I Y R E K L G N V E S
GTCTATGTCATCGATCCTGAAGATGTCGCATTGCTGTTTAAAAGTGAGGGCCCGAATCCC

V Y V I D P E D V A L L F K S E G P N P
GAACGTTTCCTGATCCCGCCGTGGGTGGCTTACCACCAATATTATCAGCGTCCAATTGGC

E R F L I P P W V A Y H Q Y Y Q R P I G
GTGCTGCTGAAGAAAAGCGCAGCTTGGAAAAAAGATCGCGTTGCCCTGAACCAAGAAGTC

V L L K K S A A W K K D R V A L N Q E V
ATGGCCCCAGAAGCAACCAAGAATTTCCTGCCTCTGCTGGATGCCGTGTCCCGCGACTTT

M A P E A T K N F L P L L D A V S R D F
GTCTCTGTTCTGCATCGTCGCATTAAGAAGGCCGGGAGCGGCAACTACAGCGGTGACATC

V S V L H R R I K K A G S G N Y S G D I
AGCGATGATCTTTTCCGTTTCGCGTTCGAATCAATTACCAACGTTATTTTTGGTGAACGT

S D D L F R F A F E S I T N V I F G E R
CAGGGCATGCTGGAGGAAGTAGTCAACCCGGAAGCGCAACGCTTCATTGATGCAATTTAC

Q G M L E E V V N P E A Q R F I D A I Y
CAGATGTTTCACACGAGCGTCCCGATGCTGAACTTACCGCCTGATCTCTTTCGCTTGTTT

Q M F H T S V P M L N L P P D L F R L F
CGTACCAAAACCTGGAAAGATCACGTTGCGGCATGGGACGTAATTTTCAGCAAAGCCGAC

R T K T W K D H V A A W D V I F S K A D
ATCTACACTCAGAATTTCTATTGGGAGCTGCGCCAGAAAGGTAGCGTACATCACGACTAT

I Y T Q N F Y W E L R Q K G S V H H D Y
CGTGGTATTCTGTATCGCCTCCTCGGTGATAGCAAGATGAGTTTTGAGGACATTAAAGCT

R G I L Y R L L G D S K M S F E D I K A
AATGTTACCGAGATGCTGGCGGGCGGTGTGGACACGACGAGCATGACTCTGCAGTGGCAT

N V T E M L A G G V D T T S M T L Q W H
TTGTACGAGATGGCGCGTAATCTGAAAGTCCAGGATATGTTACGTGCCGAAGTCCTGGCC

L Y E M A R N L K V Q D M L R A E V L A
GCCCGTCACCAAGCGCAGGGTGACATGGCAACCATGCTGCAACTGGTGCCCCTGCTGAAA

A R H Q A Q G D M A T M L Q L V P L L K
GCTAGCATCAAGGAAACGTTACGTCTGCATCCGATTAGCGTCACCTTACAGCGCTATCTG

A S I K E T L R L H P I S V T L Q R Y L
GTGAACGATCTCGTGCTCCGCGACTATATGATTCCGGCGAAAACGCTGGTTCAGGTCGCG

V N D L V L R D Y M I P A K T L V Q V A
ATTTACGCGCTGGGGCGTGAACCGACCTTCTTTTTCGATCCCGAAAACTTTGACCCGACT

I Y A L G R E P T F F F D P E N F D P T
CGCTGGTTGAGTAAAGATAAAAATATCACGTATTTTCGTAATCTTGGCTTCGGCTGGGGT

R W L S K D K N I T Y F R N L G F G W G
GTGCGCCAATGCTTAGGTCGCCGTATCGCCGAACTGGAAATGACCATTTTCCTGATTAAT

V R Q C L G R R I A E L E M T I F L I N
ATGCTGGAAAATTTCCGTGTTGAAATTCAGCATCTGTCTGATGTGGGTACCACCTTTAAT

M L E N F R V E I Q H L S D V G T T F N
CTCATTCTGATGCCGGAAAAACCTATCAGCTTCACCTTTTGGCCGTTCAACCAAGAAGCG

L I L M P E K P I S F T F W P F N Q E A ACGCAACAACACCATCACCATCACCATTAA

T Q Q H H H H H H STOP

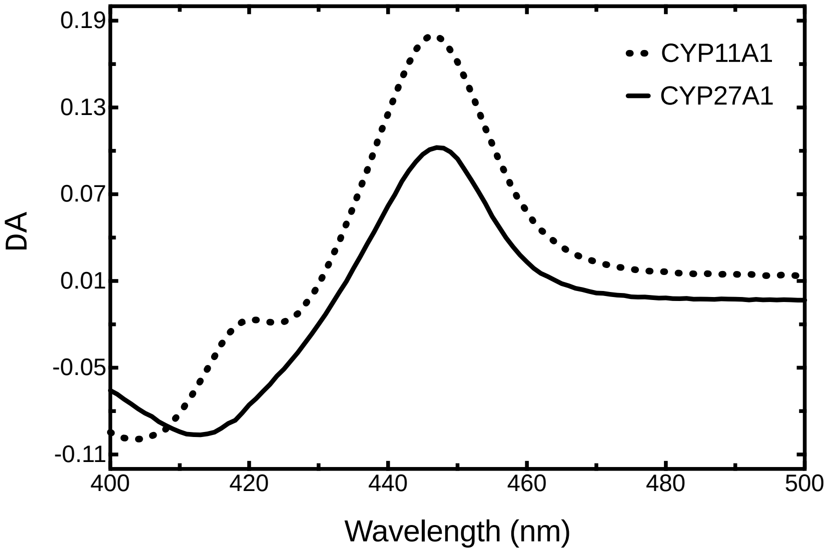

Figure S23. Fe^{2+}-CO vs. Fe^{2+} difference spectra [6] of purified CYP11A1 and CYP27A1. The concentrations of CYP11A1 and CYP27A1 were 1.8 μM and 1.1 μM respectively.

Supplementary Materials and Methods

Cholesterol synthesis network model

A substance container model was used to describe the concentrations of substances (X) by integrating in- and out-fluxes (Φ_{in} *and* Φ_{out}, respectively) (Eq. 1).

$$\frac{dX}{dt} = \phi_{in} - \phi_{out} \qquad (1)$$

The substance container model was used to model metabolites, enzymes, and mRNA concentrations. Several types of reaction models were used to describe the fluxes of reaction. To describe the reactions that define the fluxes through the metabolic network, an enzyme reaction model was used (Eq. 2).

$$\begin{aligned}
\phi_{in} &= S \cdot E \cdot k_C - C \cdot k_{CR} \\
\phi_{out} &= C \cdot k_P - P \cdot E \cdot k_{PR} \\
\frac{dC}{dt} &= \phi_{in} - \phi_{out} \\
\phi_{in} &= \phi_{inE} \\
\phi_{out} &= \phi_{outE} \\
k_{CR} &= r \cdot k_P \\
k_{PR} &= r \cdot k_C
\end{aligned} \qquad (2)$$

In Eq. 2 the symbols have the following meaning: S – concentration of substrate, E – concentration of free enzyme, P – concentration of product, C – concentration of complex, k_C – complex formation rate, k_{CR} – complex degradation rate, k_P – product formation rate, k_{PR} – product degradation rate, Φ_{inE} – enzyme utilization flux, Φ_{outE} – enzyme release flux, r – backward reaction rate ratio. For enzyme formation and degradation, linear equations were used (Eq. 3).

$$\begin{aligned}
\phi_{Eform} &= X_{mRNA} \cdot k_{Ef} \\
\phi_{Edeg} &= E \cdot k_{Ed}
\end{aligned} \qquad (3)$$

In Eq. 3 the symbols have the following meaning: Φ_{form} – enzyme formation flux, Φ_{deg} – enzyme degradation rate, X_{mRNA} – corresponding mRNA concentration, k_{Ef} – enzyme formation rate, k_{Ed} –

enzyme degradation rate. Similarly, mRNA formation and degradation fluxes were modeled (Eq. 4).

$$\phi_{Xdeg} = X_{mRNA} \cdot k_{Xd} \qquad (4)$$

In Eq. 4, Φ_{Xdeg} represents mRNA degradation flux and k_{Xd} represents mRNA degradation rate. However, mRNA formation flux was defined by the expression flux, defined either by specific substances concentration ratio or manually. As the expression levels of all the involved enzymes were measured, the manual setting of mRNA formation flux was used to specify the desired mRNA level. The metabolic network was modeled by connecting the substance container models with the corresponding reaction models. The metabolites represent the nodes of the network and the reactions connect the nodes in the network. The concentrations of all the involved substances were normalized. The values of the model parameters were set by choosing arbitrary values of the fluxes into the network and by solving the model equations for the steady-state conditions. The distribution of fluxes leaving at the network nodes were fixed according to the known network specifics and re-evaluated with simulation studies. The same values of enzyme formation fluxes were used for all enzymes; however, their degradation rates were adjusted when mRNA and corresponding enzyme levels were de-coupled. For details of the model parameters identification procedure see ref.[1]. As shown in Belic et al., when studying relative changes of substances concentrations in steady-state conditions, the choice of fluxes into the network can be arbitrarily chosen, as the parameter has no significant effect on the system amplification; however, the fluxes values strongly affect the system dynamics. The model was implemented in Dymola 5.3. (Dynasim AB, Lund, Sweden) and simulated with a Petzold integration routine (dassl).

Simulation of cholesterol synthesis pathway in the testis of *Crem*$^{-/-}$ mice: optimizing the model according to the measured genes and metabolites

The sterol regulatory element binding transcription factor 2 (SREBF2) regulation of cholesterol levels was not included in the model because gene expressions for the enzymes controlling the significant steps in the network were measured and their relative changes of *Crem*$^{-/-}$ with respect to the WT were forced on the model, replacing the simulated regulation response. The influence

of relative gene expressions on the simulated metabolite distribution changes was observed and compared to the measured values. The goal of the simulation experiments was to optimize the model in order to obtain the best accordance of the measured and the simulated metabolites (Eq. 5).

$$J = \sum_i (M_i^m - M_i^s)^2 \qquad (5)$$

In Eq. 5, J stands for criterion value, M_i^m for measured concentration of i-th metabolite, and M_i^s for simulated concentration of i-th metabolite. For optimization procedures the Nelder-Mead (simplex) method was used[2], implemented in Matlab (Mathworks, Natick, MA, USA) as *fminsearch* function. The optimization procedure (with large-scale option) was used and was repeated until no better solution could be found. The large-scale option adaptively changes the optimization step with respect to the stage of the optimization procedure. At the beginning, larger optimization steps are encouraged and, at the final stages, the optimization steps are limited. Mean squared error of simulated and measured metabolites levels was used as the criterion function.

Enzyme expression and purification

Expression and purification of CYP7A1 and CYP46A1 were described earlier[3, 4].

Expression of CYP11A1 and CYP27A1

A fresh overnight culture was prepared from *Escherichia coli* JM109 for CYP11A1 and DH5α for CYP27A1 using the plasmid, plus a plasmid containing the gene for the *E. coli* molecular chaperone GroEL/ES, to 100 mL of Luria-Bertani (LB) media containing ampicillin (100 µg/mL) and kanamycin (50 µg/mL). This starter culture was grown overnight at 37 °C and 220 rpm. Large-scale expression of CYP11A1 was done in 4.5 l (750 ml/2.8 liter flask) and CYP27A1 was done in with 6 L (1000 ml/2.8 liter flask) of Terrific Broth (TB) media supplemented with the same antibiotics (*vide supra*), bactopeptone (2 g/L), trace elements (250

μl/l of culture) [5], 1 mM NaCl, 1 mM thiamine, 4 mL of glycerol/l of culture, and an overnight starter culture (at a dilution of 1:100, v/v). Cultures were incubated for at least 6 h with shaking at 37 °C and 225 rpm, until the OD_{600} reached 0.7-0.75. The expression of CYP was induced by the addition of 1.0 mM isopropyl β-D-1-thiogalactopyranoside, 1.0 mM 5-aminolevulinic acid, and 1.0 mM and 6 mM arabinose for CYP11A1 and CYP27A1, respectively. Cultures were incubated for another 42-48 h at 28-29 °C and at 190 rpm (CYP11A1) or 150 rpm (CYP27A1) in a Multifors incubator, followed by harvesting of cells by centrifugation at 3,500 × g for 20 min.

Purification of CYP11A1 and CYP27A1

All steps were carried out at 4 °C. The recovered cell pellets (from 6 l of culture) were resuspended in 300 ml of TES buffer (100 mM Tris-acetate (pH 7.5) containing 0.5 M sucrose and 0.5 mM EDTA)/l of cell culture. Cells were mixed with lysozyme (2 mg/l of cell culture) and kept on ice for 60 min. Spheroplasts were obtained by centrifuging the cell suspension at 3,500 × *g* for 20 min. The resulting spheroplasts were resuspended in 50 ml of sonication buffer (100 mM potassium phosphate (pH 7.5) containing 16% glycerol (v/v), 9 mM magnesium acetate, 100 μM dithiothreitol, 1.0 mM phenylmethylsulfonyl fluoride (PMSF), and two protease inhibitor tablets (Roche)/L of cell culture). After sonication on ice, the material was centrifuged at 12,000 × *g* for 15 min, and the recovered supernatant was further centrifuged at 140,000 × *g* for 60 min. The pellet obtained after ultracentrifugation was collected, homogenized, and solubilized in 600 mL of solubilization buffer (100 mM potassium phosphate buffer (pH 7.5) containing 20% glycerol (v/v), 0.1 mM EDTA, 10 mM *β*-mercaptoethanol, 0.5 M KCl, and 1.0% CHAPS (w/v, for CYP11A1) or 1.0% sodium cholate (w/v, for CYP27A1)). The resulting material was centrifuged at 140,000 × *g* for 60 min, and the supernatant was loaded onto a 1.5 × 8 cm Ni^{2+}-NTA (nitrilotriacetic acid) column, which had been pre-equilibrated with 100 mM potassium phosphate buffer (pH 7.5) containing 20% glycerol (v/v), 10 mM *β*-mercaptethanol, 0.5 M KCl, 1.0% CHAPS (w/v, for CYP11A1) or 1.0% sodium cholate (w/v, for CYP27A1). The column was washed with the same equilibration buffer containing 20 mM imidazole for CYP27A1 (CYP11A1 was washed with equilibration buffer containing 2.8 mM C8E5 detergent along with 20 mM imidazole). CYP27A1 was eluted with the buffer containing 200 mM imidazole (other compositions were kept the same) and CYP11A1 was eluted with 200 mM imidazole containing 2.8 mM C8E5 detergent (other compositions were kept the same). The

eluted fractions containing CYP were dialyzed against 2 L of 100 mM potassium phosphate buffer (pH 7.5) containing 20% glycerol (v/v, 24 h, three buffer changes). The dialyzed protein was stored at -20 ^{o}C until further use.

The concentration of CYP was measured spectrally[6] using an OLIS/Aminco DW2 spectrophotometer (On-Line Equipment Systems, Bogart, GA, USA). Typical yields of purified protein were 135 and 400 nmol for CYP11A1 and CYP27A1, respectively.

Expression and purification of ADX and ADR

Bovine ADX was expressed in *E. coli* DH5α cells with the expression plasmid pBA1159[7]. A single colony was grown overnight in LB media with 100 μg/ml ampicillin at 37 °C with gyrorotary shaking at 220 rpm. An overnight culture (5 ml) was then inoculated into 500 mL of TB media containing 100 μg/ml ampicillin, 340 μg/ml thiamine, and 0.025% (v/v) of a mixture of trace elements[5]. The cultures were incubated at 37 °C with gyrorotary shaking at 220 rpm for 4 hours, and ADX expression was induced by the addition of 1.0 mM IPTG. The incubation continued at 30 °C for another 24 h. Cells were harvested by centrifugation at 3,000 × g for 5 min, and the cell pellet was sonicated in 10 mM potassium phosphate buffer (pH 7.4). Cell debris was removed by centrifugation at 100,000 × g for 30 min, and the supernatant was used for further purification by DEAE-cellulose and G-75 Sephadex chromatography.

The above supernatant was applied directly to a 2.5 cm × 20 cm column of DEAE-Sepharose equilibrated with 50 mM potassium phosphate buffer (pH 7.4) containing 0.1 mM EDTA and washed with 1 l of the equilibration buffer. The column was eluted with the same buffer containing 170 mM NaCl and then 300 mM NaCl Those fractions showing absorbance at 414 nm from the 170 mM pool were highly purified as judged by SDS-PAGE (15% (w/v) gel). The fractions eluted with 300 mM NaCl were dialyzed twice vs. 10 mM potassium phosphate buffer (pH 7.4) containing 0.1 mM EDTA and applied to a 2.5 × 10 cm column of DEAE-Sepharose, which was washed with the dialysis buffer and then eluted with the same containing 500 mM KCl. Fractions containing Adx were pooled on the basis of A_{414} measurements. Fractions from the DEAE steps were further purified with gel filtration chromatography on a 2.5 × 100 cm column of Sephadex G-75, using 10 mM potassium phosphate buffer (pH 7.4) containing 0.1

mM EDTA (≤ 20 mL per run). The total yield was 25,700 nmol Adx, which was stored in aliquots at – 70° C.

Bovine ADR was expressed in *E. coli* JM109 cells as described[8], in a pCWori$^+$ expression vector[9] with a $(His)_6$ tag added to the C-terminal of the cDNA to facilitate protein purification. In brief, a single colony was picked and grown overnight in LB media with 100 µg/ml ampicillin at 37 °C, with gyrorotary shaking at 220 rpm. Five mL of overnight culture was then inoculated into 500 mL of TB media containing 100 µg/mL ampicillin, 340 µg/mL thiamine, and 0.025% (v/v) of a mixture of trace elements[5]. The cultures were incubated at 37 °C with gyrorotary shaking at 220 rpm for 4 h, and ADR expression was induced with the addition of 1.0 mM IPTG. The incubation continued at 30 °C for another 24 h. The purification was conducted with a Ni^{2+}-NTA (nitrilotriacetic acid) column. Briefly, cells were harvested by centrifugation at 3,000 × g for 10 min and cell pellet was sonicated in 100 mM potassium phosphate (pH 7.4) containing 0.1 mM EDTA, 20% glycerol (v/v), and protease inhibitor tablets (Roche). Cell debris was removed by centrifugation at 100,000 × g for 30 min and the supernatant was loaded on the Ni^{2+}-NTA column. The column was washed with same buffer containing 20 mM imidazole, and ADR was eluted with the same buffer containing 200 mM imidazole. The eluted ADR was dialyzed against 40 volumes of 100 mM potassium phosphate buffer (pH 7.4) containing 20% glycerol (v/v) and 0.1 mM EDTA three times to remove imidazole.

Sterols and other reagents

7-Dehydrocholesterol, cholesterol, 2-hydroxypropyl-*β*-cyclodextrin (HP*β*CD), *N*,*O*-bis(trimethylsilyl) trifluoroacetamide (BSTFA), L-*α*-1,2-dilauroyl-*sn*-glycero-3-phosphocholine, and pregnenolone were purchased from Sigma-Aldrich. Lanosterol, dihydrolanosterol, FF-MAS, T-MAS, zymosterol, 7-dehydrodesmosterol, zymostenol, lathosterol, desmosterol, and 7*α*-hydroxycholesterol were from Avanti Polar Lipids (Alabaster, AL). 24-Hydroxycholesterol was obtained from AbcamBiochemicals (Cambridge, UK), 25-hydroxycholesterol was from Cayman Chemicals (Ann Arbor, MI), and 27-hydroxycholesterol was from Enzo Life Sciences. All organic solvents (Fisher) were HPLC grade.

Enzyme activity assays

Enzymatic assays were carried out in a 0.5 mL reaction volume containing CYP, NADPH-P450 reductase (1:2 molar ratio for CYP46A1 and 1:10 molar ratio for CYP7A1) or ADX and ADR (1:2:0.2 molar ratios for CYP27A1 and CYP11A1), 100 mM potassium phosphate buffer (pH 7.5), 150 μM l-α-1,2-dilauroyl-*sn*-glycero-3-phosphocholine, and sterol substrate which was initially dissolved in 310 mM HPβCD (45% HPβCD (w/v)). A 5-μl aliquot of stock substrate solution was added to the 500 μl final reaction volume. After pre-incubation at 37 $^{\circ}$C, enzymatic reactions were initiated by the addition of 150 μl of an NADPH-generating system[10] and incubated at 37 $^{\circ}$C. The reaction was quenched with CH_2Cl_2 (2 ml), and the products were extracted twice with 2 ml of CH_2Cl_2, following centrifugation at 2,000 × *g* for 10 min (23 °C) to separate the layers. The organic layers were combined and dried under a nitrogen stream. The dried samples were dissolved in CH_3CN and subjected to LC-MS analysis. LC-MS was performed using a Waters Acquity UPLC system and ACQUITY UPLC BEH octadecylsilane (C_{18}) column (1.7 μm, 2.1 mm × 100 mm) at 35 $^{\circ}$C with a flow rate of 0.3 ml/min. Mobile phase A consisted of 95:5 (v/v) H_2O and CH_3CN with 0.1% HCO_2H (v/v); mobile phase B consisted of 5:95 (v/v) H_2O and CH_3CN with 0.1% HCO_2H (v/v). For CYP11A1, CYP27A1, and CYP46A1, the LC program was as follows: 0-8 min: 80% mobile phase B (v/v), 8-9 min: 80% mobile phase B to 100% mobile phase B (v/v), 9-20 min: 100% mobile phase B, 20-21 min: 100% mobile phase B to 80% mobile phase B (v/v), 21-30 min: 80% mobile phase B (v/v). For CYP7A1, the LC method was as follows: 0-8 min: 85% mobile phase B (v/v), 8-9 min: 85% mobile phase B to 100% mobile phase B (v/v), 9-20 min: 100% mobile phase B, 20-21 min: 100% mobile phase B to 85% mobile phase B (v/v), 21-30 min: 85% mobile phase B (v/v).

Sterol separation and identification

LC-MS was done using a Waters Acquity UPLC system connected to a Thermo LTQ mass spectrometer. Analyte (10 µl, unless stated otherwise) was injected into the system in the needle overfill mode (otherwise stated). An APCI$^+$ source was used in all cases with the following parameters: *For 7α-hydroxycholesterol*: discharge voltage, 3.9 kV; discharge current, 5 μA; vaporizer temperature, 450 °C; sheath gas flow rate, 50 arbitrary units; auxiliary gas flow rate, 5

arbitrary units; sweep gas flow rate, 5 arbitrary units; capillary voltage, 4.75 V; capillary temperature, 275 °C; tube lens voltage, 50 V. *For pregnenolone*: discharge voltage, 3.8 kV; discharge current, 5 μA; vaporizer temperature, 450 °C; sheath gas flow rate, 50 arbitrary units; auxiliary gas flow rate, 5 arbitrary units; sweep gas flow rate, 5 arbitrary units; capillary voltage, 14 V; capillary temperature, 275 °C; and tube lens voltage, 65 V. *For 27-hydroxycholesterol*: discharge voltage, 4 kV; discharge current, 5 μA; vaporizer temperature, 450 °C; sheath gas flow rate, 50 arbitrary units; auxiliary gas flow rate, 5 arbitrary units; sweep gas flow rate, 5 arbitrary units; capillary voltage, 30 V; capillary temperature, 275 °C; tube lens voltage, 85 V. *For 24-hydroxycholesterol*: 4.1 kV; discharge current, 5.1 μA; vaporizer temperature, 450 °C; sheath gas flow rate, 50 arbitrary units; auxiliary gas flow rate, 5 arbitrary units; sweep gas flow rate, 5 arbitrary units; capillary voltage, 18 V; capillary temperature, 275 °C; tube lens voltage, 80 V.

GC-MS analysis was performed using a gas chromatograph (Agilent 6890 GC) coupled with a mass selective detector (Agilent MSD 5973). An Agilent capillary column was used (DB-5; 30 m length, 0.25 mm i.d., 0.25 μm film thickness). Helium was used as the carrier gas, maintained at a constant flow rate of 1.5 ml/min. The GC temperature program was as follows: initial temperature 70 °C for 1 min; program from 70 to 130 °C at a rate of 50 °C/min, hold for 1 min at 130 °C; program from 130 to 300 °C at a rate of 15 °C/min, hold at 300 °C for 15 min. Analyte was injected in a splitless mode. MS conditions were as follows: electron impact[11] mode at an ionization energy of 70 eV, transfer line and ion source temperature at 280 and 230 °C, respectively. Mass spectra were recorded from *m/z* 50-700.

A 600 MHz Bruker NMR spectrometer was used for acquiring NMR spectra. The NMR spectrometer was equipped with a 5-mm Z-gradient TCI cryoprobe, and Topspin software was used to analyze the data. $CDCl_3$ was used as a solvent, and the peak at (δ) 7.26 ppm for $CHCl_3$ was used as a reference.

Sample preparation for GC-MS analysis

Enzymatic assays with CYP46A1 and sterols (zymostenol and lathosterol) were carried out in a 5 mL reaction volume containing 2 μM CYP, 4 μM NADPH-P450 reductase, 100 mM potassium phosphate buffer (pH 7.5), 150 μM L-α-1,2-dilauroyl-*sn*-glycero-3-phosphocholine,

and 50 μM substrate (which was initially dissolved in 310 mM HPβCD (45% HPβCD (w/v)), incubated, extracted, and dried as described above. The oxidative products obtained from enzymatic assay were first purified before converting in to TMS ether derivatives for GC-MS analysis. Purification was done on a Waters Acquity UPLC system and using an ACQUITY UPLC BEH octadecylsilane (C_{18}), 1.7 μm, 2.1 mm ×100 mm column at 35 °C with a flow rate of 0.6 mL/min. Samples (20 μl) were injected into the system in a partial loop mode. The LC-MS profile was used for collection, with a 8:2 (v/v) split into the mass spectrometer. Mobile phase A consisted of 95:5 (v/v) H_2O and CH_3CN with 0.1% HCO_2H (v/v); mobile phase B consisted of 5:95 (v/v) H_2O and CH_3CN with 0.1% HCO_2H (v/v). The LC method was as follows: 0-7.0 min: 65% mobile phase B (v/v), 7.0-7.5 min: 65% mobile phase B (v/v) to 100% mobile phase B, 7.5-14.5 min: 100% mobile phase B, 14.5-15.0 min: 100% mobile phase B to 65% mobile phase B (v/v), 15.0-20.0 min: 65% mobile phase B (v/v). Purified fractions were collected and combined, extracted into CH_2Cl_2, dried under nitrogen. The purified products were converted into trimethylsilyl (TMS) ether derivatives. Derivatization was done using BSTFA:dimethylformamide (1:1 ratio, v:v) at 23 °C for 60 min, and samples were directly subjected to GC-MS analysis. The fragmentation patterns of TMS ether derivatives of the products were analyzed for structure elucidation.

The use of TMS ethers for positional analysis is a well-established procedure for hydroxyl groups on fatty acids, sterols, and other aliphatic molecules[12-14] and has been utilized extensively in this laboratory[15-17]. Treatment of an alcohol with BSTFA yields a TMS ether at a hydroxyl group. Fragmentation (in GC/MS, using the electron impact mode) breaks the bonds on either side of the ether, and the loss of a fragment yields a product with an m/z characteristic of the loss of a 1, 2, 3,…carbon unit. This strategy[12-17] is very applicable to the side chains of sterols. See Fig. 6 (body of paper). The fragment *m/z* 131 is indicative of loss of the elements of $C(CH_3)_2OSi(CH_3)_3$, consistent with hydroxylation at C25 (Fig. 6, parts 2,4A). The fragment *m/z* 145 (addition of one methylene) is indicative of loss of the elements of $(CH_3)_2CH_2COSi(CH_3)_3$, consistent with hydroxylation at C24 (part 4B). These fragmentation patterns (base peaks) were matched with standard 24- and 25-hydroxycholesterol (data not shown) with the assumption that a change in the position of double bond in “B” ring of sterols would not have any effect on the α-cleavage of an TMS isopropyl ether of the side chain, which is far from the "B" ring of the sterol.

Sample preparation for NMR analysis

Large-scale incubations were done to obtain sufficient amounts of products for NMR analysis. Enzymatic assays with CYP27A1 (50 nmol CYP27A1, 100 nmol Adx, and 10 nmol ADR) and CYP7A1 (50 nmol CYP7A1, 250 nmol NADPH-P450 reductase) and sterols (zymostenol, lathosterol, 7-dehydrocholesterol, and desmosterol, respectively) were carried out in a 50-mL reaction volume, including 100 mM potassium phosphate buffer (pH 7.5), 150 μM L-α-1,2-dilauroyl-*sn*-glycero-3-phosphocholine, and 50 μM substrate (which was initially dissolved in 310 mM HPβCD (45% HPβCD (w/v)). After pre-incubation at 37 ^{o}C, reaction was started by addition of 15 mL of an NADPH-generating system [10] and further incubated at 37 ^{o}C for 2 h, extracted (with 160 mL of CH_2Cl_2) and dried as described above. The dried sample was dissolved in CH_3OH, centrifuged (2,000 × g, 10 min) to precipitate suspended particles. Purification was done using Waters Acquity UPLC system, in that there was not useful UV chromophore, and using an ACQUITY UPLC BEH octadecylsilane (C_{18}) column (1.7 μm, 2.1 mm ×100 mm) at 35 $^{\circ}$C with a flow rate of 0.6 mL/min. Samples (20 μL) were injected into the system using a partial loop mode. The LC-MS profile was used for collection, with a 8:2 (v/v) split into the mass spectrometer. Mobile phase A consisted of 95:5 (v/v) H_2O and CH_3CN with 0.1% HCO_2H (v/v); mobile phase B consisted of 5:95 (v/v) H_2O and CH_3CN with 0.1% HCO_2H (v/v). The LC method was as follows: 0-7.0 min: 65% mobile phase B (v/v), 7.0-7.5 min: 65% mobile phase B (v/v) to 100% mobile phase B, 7.5-14.5 min: 100% mobile phase B, 14.5-15.0 min: 100% mobile phase B to 65% mobile phase B (v/v), 15.0-20.0 min: 65% mobile phase B (v/v). Purified fractions were combined, extracted into CH_2Cl_2, dried under nitrogen, and re-dissolved in $CDCl_3$ for NMR analysis.

References

1. Belic, A., Acimovic, J., Naik, A. & Golicnik, M. Analysis of the steady-state relations and control-algorithm characterisation in a mathematical model of cholesterol biosynthesis. *Simul Model Pract Th* **33**, 18-27 (2013).
2. Nelder, J.A. & Mead, R. A simplex method for function minimization. *The Computer Journal* **7**, 308–313 (1965).
3. Shinkyo, R. & Guengerich, F.P. Cytochrome P450 7A1 cholesterol 7alpha-hydroxylation: individual reaction steps in the catalytic cycle and rate-limiting ferric iron reduction. *The Journal of biological chemistry* **286**, 4632-4643 (2011).
4. Goyal, S., Xiao, Y., Porter, N.A., Xu, L. & Guengerich, F.P. Oxidation of 7-dehydrocholesterol and desmosterol by human cytochrome P450 46A1. *Journal of lipid research* **55**, 1933-1943 (2014).
5. Sandhu, P., Baba, T. & Guengerich, F.P. Expression of modified cytochrome P450 2C10 (2C9) in Escherichia coli, purification, and reconstitution of catalytic activity. *Archives of biochemistry and biophysics* **306**, 443-450 (1993).
6. Omura, T. & Sato, R. The Carbon Monoxide-Binding Pigment of Liver Microsomes. I. Evidence for Its Hemoprotein Nature. *The Journal of biological chemistry* **239**, 2370-2378 (1964).
7. Sagara, Y. et al. Direct expression in Escherichia coli and characterization of bovine adrenodoxins with modified amino-terminal regions. *FEBS letters* **300**, 208-212 (1992).
8. Sagara, Y. et al. Direct expression of adrenodoxin reductase in Escherichia coli and the functional characterization. *Biological & pharmaceutical bulletin* **16**, 627-630 (1993).
9. Barnes, H.J., Arlotto, M.P. & Waterman, M.R. Expression and enzymatic activity of recombinant cytochrome P450 17 alpha-hydroxylase in Escherichia coli. *Proceedings of the National Academy of Sciences of the United States of America* **88**, 5597-5601 (1991).
10. Guengerich, F.P. in Hayes' Principles and Methods of Toxicology, 6th Edn. (eds. A.W. Hayes & C.L. Kruger) CRC Press, Boca Raton, FL, USA (2014).
11. Hanna, I.H., Teiber, J.F., Kokones, K.L. & Hollenberg, P.F. Role of the alanine at position 363 of cytochrome P450 2B2 in influencing the NADPH- and hydroperoxide-supported activities. *Archives of biochemistry and biophysics* **350**, 324-332 (1998).
12. Perkins, E.G. & Argoudelis, C.J. Determination of double bond position in polyunsaturated fatty acids using combination gas chromatography mass spectrometry. *Lipids* **4**, 619-621 (1969).
13. Wheelan, P., Zirrolli, J.A. & Murphy, R.C. Analysis of hydroxy fatty acids as pentafluorobenzyl ester, trimethylsilyl ether derivatives by electron ionization gas chromatography/mass spectrometry. *Journal of the American Society for Mass Spectrometry* **6**, 40-51 (1995).
14. Wood, K.V., Bonham, C.C. & Jenks, M.A. The effect of water on the ion trap analysis of trimethylsilyl derivatives of long-chain fatty acids and alcohols. *Rapid communications in mass spectrometry : RCM* **15**, 873-877 (2001).
15. Chun, Y.J. et al. Electron transport pathway for a Streptomyces cytochrome P450: cytochrome P450 105D5-catalyzed fatty acid hydroxylation in Streptomyces coelicolor A3(2). *The Journal of biological chemistry* **282**, 17486-17500 (2007).
16. Tang, Z., Martin, M.V. & Guengerich, F.P. Elucidation of functions of human cytochrome P450 enzymes: identification of endogenous substrates in tissue extracts using metabolomic and isotopic labeling approaches. *Anal Chem* **81**, 3071-3078 (2009).
17. Xiao, Y. & Guengerich, F.P. Metabolomic analysis and identification of a role for the orphan human cytochrome P450 2W1 in selective oxidation of lysophospholipids. *Journal of lipid research* **53**, 1610-1617 (2012).
